# Shuttle-box systems for studying preferred environmental ranges by aquatic animals

**DOI:** 10.1093/conphys/coab028

**Published:** 2021-05-17

**Authors:** Emil A F Christensen, Lars E J Andersen, Heiðrikur Bergsson, John F Steffensen, Shaun S Killen

**Affiliations:** 1 Institute of Biodiversity, Animal Health and Comparative Medicine, University of Glasgow, 82 Hillhead Street, Glasgow, G12 8QQ, UK; 2 Marine Biological Section, University of Copenhagen, Strandpromenaden 5, 3000 Elsinore, Denmark

**Keywords:** Avoidance, behaviour, eco-physiology, preference

## Abstract

**Animals’ selection of environments within a preferred range is key to understanding their habitat selection, tolerance to stressors and responses to environmental change. For aquatic animals, preferred environmental ranges can be studied in so-called shuttle-boxes, where an animal can choose its ambient environment by shuttling between separate choice chambers with differences in an environmental variable. Over time, researchers have refined the shuttle-box technology and applied them in many different research contexts, and we here review the use of shuttle-boxes as a research tool with aquatic animals over the past 50 years. Most studies on the methodology have been published in the latest decade, probably due to an increasing research interest in the effects of environmental change, which underlines the current popularity of the system. The shuttle-box has been applied to a wide range of research topics with regards to preferred ranges of temperature, CO**_**2**_ **, salinity and O**_**2**_  **in a vast diversity of species, showing broad applicability for the system. We have synthesized the current state-of-the-art of the methodology and provided best practice guidelines with regards to setup, data analyses, experimental design and study reporting. We have also identified a series of knowledge gaps, which can and should be addressed in future studies. We conclude with suggesting some obvious directions for research using shuttle-boxes within evolutionary biology and behavioural and physiological ecology**.

## Teaser

Preferred environmental ranges of aquatic animals can be estimated in so-called shuttle boxes. Here, we review the past 50 years use of shuttle boxes and synthesize the current methodological state-of-art. We also suggest directions for future studies.

## Introduction

Motile organisms can actively choose environments that are physiologically favourable and avoid those that are averse (Dillon *et al.*, 2010; Sunday *et al.*, 2014). Selection of environments within a preferred range can therefore be viewed as behavioural manifestations of animals’ physiological response to their environment (Huey, 1991). The preferred environmental ranges by animals have been studied within a range of research fields, including evolutionary biology (Angilletta *et al.*, 2002; Pilakouta *et al.*, 2019), ecology (Martin and Huey, 2008; Sunday *et al.*, 2014) and animal physiology (Fry, 1971; Huey *et al.*, 2012; Pinsky *et al.*, 2019). Recently, preferred environmental ranges by animals have also been incorporated in modelling of species distribution and responses to environmental change (Kearney *et al.*, 2009; Huey *et al.*, 2012; Pinsky *et al.*, 2019). Although most studies on preferred environmental ranges have been on terrestrial animals, there is currently a growing interest in studying this in aquatic animals (Jutfelt *et al.*, 2017).

Environmental preference is defined as the environmental level that is most frequently occupied by an animal in a free-choice situation (Reynolds and Casterlin, 1979a), while environmental avoidance is defined as the incipient level along an environmental gradient an animal will start to actively move away from (Ern, 2019). Environmental preference and avoidance can be studied in laboratory settings with the basic idea of presenting an animal with an environmental gradient and assessing its choice. The benefit of controlled laboratory experiments is that environmental preference and avoidance levels can be determined while excluding potential confounding factors, while the drawback is that studying interactive effects of multiple biotic and abiotic determining factors becomes difficult. However, with the appropriate modifications it is possible to conduct controlled experiments with multiple factors to investigate how they interact and affect preferred environmental ranges (e.g. Schurmann *et al.*, 1991; Nielsen and McGaw, 2016; Tietze and Gerald, 2016; Cooper *et al.*, 2018).

**Figure 1:**
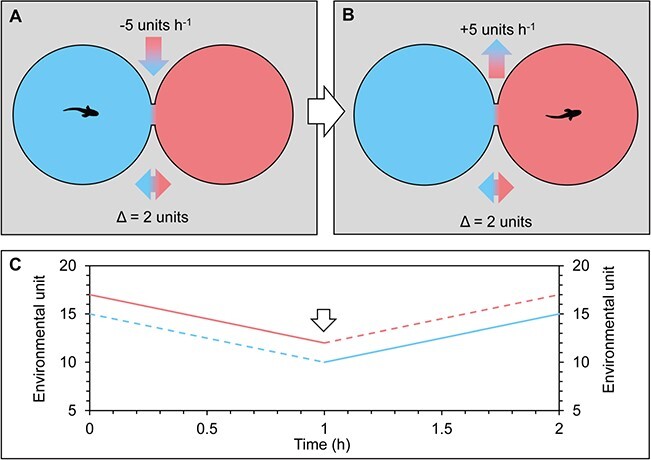
Diagram of the shuttle-box technology for studying preferred environmental ranges by aquatic animals (exemplified with a fish). A constant environmental difference (Δ) between the choice chambers can be maintained and the environmental variable changed according to the position of the animal. (**A**) shows a ‘dynamic’ system when the animal is present in the choice chamber with the lower level of the environmental variable (blue), while (**B**) shows the system if the animal changes position (white arrow) to the choice chamber with the higher level of the environmental variable (red). (**C**) provides an overview of the change in the environmental variable in both choice chambers over time, where dashed lines indicate presence of the animal at a given time.

A range of setups have been developed for experimental studies of preferred environmental ranges by aquatic animals, which each have their inherent limitations. For instance, temperature and salinity gradients can be obtained by vertical stratification (Fritz and Garside, 1974; Lafrance *et al.*, 2005). However, maintenance of a vertical gradient is not possible with environmental variables that do not stratify (e.g. environmental gasses). Other systems maintain horizontal gradients in a continuous body of water, e.g. by pointwise water treatment in either linear or annular systems (Myrick *et al.*, 2004; Wallman and Bennett, 2006), by separating different water bodies with laminar flows (Jutfelt *et al.*, 2017). However, in systems with a continuous body of water differences in water densities may cause vertical stratification of the water (Jutfelt *et al.*, 2017). Furthermore, the water may be mixed horizontally by animal movement, which may render subsequent occurrence analyses imprecise. Issues with unwanted water mixing and stratification can be reduced significantly by having physically separated choice chambers that are interconnected by narrow passages for the animal, such as so-called shuttle-boxes, which provides stable water separation for a variety of environmental variables (Schurmann *et al.*, 1991; Serrano *et al.*, 2010; Kates *et al.*, 2012; Borowiec *et al.*, 2018).

The term ‘shuttle-box’ was first used to describe a system for studying preferred temperature of aquatic animals that consisted of two physically separated, but interconnected, choice chambers between which a temperature difference was maintained (Neill *et al.*, 1972). Uniquely for their system, an animal’s presence in the warmer choice chamber automatically activated heating of the whole system, while the animal’s presence in the cooler choice chamber activated cooling of the whole system (Fig. 1). The animal thus constantly had the choice between two different temperatures, and the change in temperature setting according to the position of the animal would ultimately act as incentive for the animal to shuttle between the chambers when the temperature went outside its preferred range. Although the behaviour studied in such shuttle-boxes is, in essence, conditioned, and not innate (Wallman and Bennett, 2006), it has been used with a range of different animal species and taxa. In later years, the term ‘shuttle-box’ has become synonymous with a system that also consists of two physically separated choice chambers interconnected via a small passage but where one or both of the choice chambers are maintained at a static level (e.g. Kates *et al.* 2012; Tix *et al.* 2018). We have therefore included studies with both usages of the term in the present review and differentiate between them as ‘dynamic shuttle-boxes’ and ‘static shuttle-boxes’.

The versatility of the shuttle-box is probably the reason the system is one of the most used set ups for examining preferred environmental ranges by aquatic animals (Wallman and Bennett, 2006). Over time, the shuttle-box has been redeveloped significantly both with respect to basic set up and experimental application (e.g. Reynolds, 1977; Schurmann and Christiansen, 1994; Serrano *et al.*, 2010; Herbert *et al.*, 2012; Kates *et al.*, 2012; Cooper *et al.*, 2018). The many different directions of use of the system may affect repeatability and comparability of studies, and we have therefore systematically reviewed the use of shuttle-boxes for determining environmental preference and avoidance by aquatic animals. The present review goes through the historical use of shuttle-boxes over the past 50 years; describe the current state-of-the-art with regards to setup, data analyses and experimental design, and study reporting; and highlight directions for future studies.

## Literature review

The literature search was conducted using Google Scholar. We initially performed a search based on terms used to describe shuttle-box systems in our own published studies (Schurmann et al., 1991; Schurmann and Steffensen, 1992; Petersen and Steffensen, 2003; Killen, 2014; Nay et al., 2015, 2020; Habary et al., 2016; Christensen and Grosell, 2018; Nati et al., 2018; Pilakouta et al., 2019; Christensen et al., 2021; Christensen et al., 2020). The list of search terms was extended each time we encountered a new synonym for a shuttle-box (e.g. ‘ichthyotron’; Reynolds 1977), a new type of animal used or a new environmental variable used (summarized in Table 1). We only included studies that had provided experimental examination of preferred environmental ranges by aquatic animals, in relation to a physico-chemical variable using a shuttle-box system, which excluded studies on animal activity only, and shock behaviour in psychology studies (e.g. Bachman *et al.*, 1979; Pather and Gerlai, 2009). The studies that fulfilled our criteria were systematically checked for more references to studies using shuttle-box systems in their respective methods sections. Furthermore, we used the ‘cited by’ function in Google Scholar to find shuttle-box studies citing the already discovered literature. We also only used published literature, and not ‘grey literature’, such as Master’s theses, as this may have created biases from our own research groups. Based on this literature search, we assembled a database (supplementary material; ‘Shuttle-box database’). For each study, we noted information about what environmental variable was examined, the study species and their phylogenetic classification, information on sample size and body size of animals, acclimation conditions, system properties, experimental methodology and experimental results.

**Table 1:** List of search terms used in the systematic literature review

Primary search term	And	And/or
Shuttlebox Shuttle-box Shuttle box Two chamber choice tank Electronic shuttlebox Electronic shuttle-box Ichthyotron	Fish Shark Invertebrate Crab Lobster Shrimp Crayfish	Preference Behavior Temperature Salinity Oxygen Carbon dioxide O_2_ CO_2_ Environmental preference Temperature preference Preferred temperature Behavioral thermoregulation Salinity preference Preferred salinity Oxygen Preferred oxygen level Avoidance Environmental avoidance Temperature avoidance Salinity avoidance Oxygen avoidance Carbon dioxide avoidance O_2_ avoidance CO_2_ avoidance Chemical agent Hydrogen sulfide

## Historical use of shuttle-box systems

We found a total of 76 studies that used shuttle-boxes to examine preferred environmental ranges by aquatic animals, most of which (55) were conducted in dynamic shuttle-boxes (Table 2). Most studies have examined behavioural thermoregulation, but shuttle-boxes have also been used to study environmental preference to ambient CO_2_, salinity and O_2_ levels. Furthermore, 49 studies used additional experimental factors-both biotic and abiotic (Table 2). These findings demonstrate the versatility of shuttle-boxes for studying preferred environmental ranges of aquatic animals.

**Table 2:** Numbers of published studies using shuttle-boxes for determining environmental preference range by aquatic animals and how many of them used additional experimental factors

Environmental variable	Temperature	CO_2_	O_2_	Salinity	Total
Shuttle-box methodology					
Dynamic	51^a^		1	3	55^a^
Static	7^a^	8	5	2	22^a^
Total	58^a^	8	6	5	76^a^
Additional experimental factor					
Acclimation	10	1		2	
Acute hypoxia	4				
Anaemia			1		
Animal size	2				
Blood haemoglobin type	1				
Endogen cortisol level		1			
Feeding (during trial)	1			1	
Feeding regime (growth trajectory, feed/fasted, and food type)	1	1		1	
Food trade-off	1				
Group assay	1				
HSO_4_ exposure	1				
Infection					
Light level	3	1			
Nitrate level			1		
Ontogenetic shift				1	
Population differences	5				
Predator trade-off				1	
Seasonality	1				
Shelter and structural environment trade-off	2				
Social hierarchy	1				
Sociality	2		1		
Total	36	4	3	6	48

a ^a^Note that Cooper et al. (2018) used their temperature shuttle-box both dynamically and statically.

**Figure 2:**
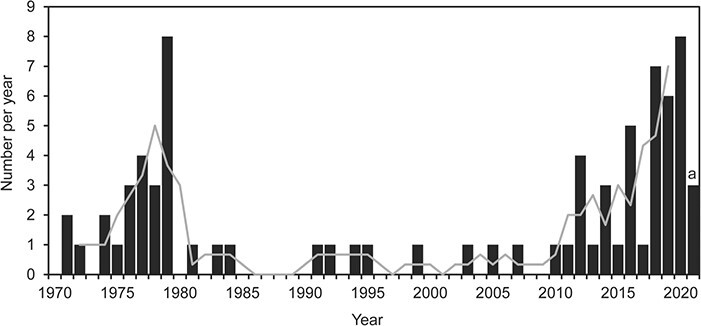
Number of publications per year of studies using the shuttle-box system for studying preferred environmental ranges by aquatic animals (black bars). ^a^Only represent numbers at the start of March 2021. The grey line represents the weighed trend of a 3-year running mean, centered around any given year, excluding 2021.

Many of the shuttle-box studies were published in the 1970s (24) and 2010s (31; Fig. 2). The recent increase in numbers of published studies is probably due to an increasing research interest in the effects of environmental change, and highlights the current popularity of the shuttle-box system. The publications in the 1970s originated from a few research groups, while more recent publications have been conducted in a wider variety of research groups. Furthermore, all studies before 2010 examined behavioural thermoregulation in different species, while, more recently, shuttle-boxes have begun to be used for also assessing preferences for additional environmental variables.

**Table 3:** Taxonomic distribution of the aquatic animals used in shuttle-box studies on preferred environmental ranges

Taxonomic level	Phylum	Sub-phylum	Class	Order	Family	Genus	Species
Chordates	1	1	3				
Ray-finned fish				15	31	45	55
Elasmobranchs				2	2	2	2
Lampreys				1	1	1	1
Crustaceans	1	2					
Decapods			1	1	4	6	6
Cheliceratids			1	1	1	1	1
Total	2	3	5	20	39	55	65
Habitat							
Freshwater species	2	2	3	14	18	29	38
Marine species	2	3	4	10	19	22	24
Euryhaline species	1	1	1	2	3	3	3
Total							64

**Table:** 

Summary box for: ‘historical use of shuttle-boxes’	
Element	Synthesis
Publications numbers	A total of 76 published studies, with most per decade in the 2010s
Environmental variables	Used to study preferred environmental ranges of temperature, CO_2_, salinity and O_2_
Methodology	Mostly in dynamic shuttle-boxes 49 studies used and interactive experimental factor in addition to the environmental factor
Taxonomy	Used in 65 species from a variety of taxa Mostly used in freshwater species
Identified knowledge gaps Phylogenetic studies on evolution of preferred environmental ranges Effects of species lifestyle on preferred environmental range Few studies on non-ray finned fish Few studies on invertebrates	

Shuttle-box experiments have been conducted on a total of 65 aquatic species, of which the vast majority are ray-finned fishes (55; Table 3). Most species examined have been freshwater species (37). Within the bony fishes, the study species have covered a wide range of taxa with 15 orders, 31 families and 45 genera. Notably, to date there are no studies that investigate trends across multiple species, for instance to examine the evolution of environmental preference and avoidance, or the effects of species lifestyle. Being compatible with many different species and taxa, shuttle-box studies would be suitable for studying the question of the evolution of preferred environmental ranges as well as more for studies on non-ray-finned fishes and invertebrates.

## Shuttle-box systems

We will here describe the current state-of-the-art of shuttle-box design and use. A detailed instruction for how to build and set up shuttle-boxes can be found in the supplementary materials (`How to build and set up shuttle-boxes').

### Physical appearance

Modern shuttle-boxes consist of two, rounded choice chambers (see Figure S1 in the supplementary material). Early versions of shuttle-boxes had square choice chambers, and, to our knowledge, the first published study using circular choice chambers was Petersen and Steffensen (2003). Circular choice chambers prevent animals from using corners as ‘hiding places’, which can otherwise happen in square shuttle-boxes (Bevelhimer, 1996; Reiser *et al.*, 2013). Furthermore, having oppositely directed circular water currents in circular choice chambers enables the waterflow along the passage between the choice chambers to be concurrent, and not counter current, which creates an effective barrier for water mixing. Most studies published after Petersen and Steffensen (2003) used circular chambers, except for Tattersall *et al.* (2012) and Skandalis et al. (2020). Most shuttle-boxes since Schurmann *et al.* (1991) have used mixing chambers for water treatment (aeration and environmental regulation) and water mixing, except Tattersall *et al.* (2012) and Skandalis *et al.* (2020). Using mixing chambers eliminates undesired gradients within each choice chamber, which can occur if environmental regulation is carried out directly within the choice chambers.

Shuttle-boxes can, in principle, consist of more than two choice chambers. An elegant example is the four-chamber dynamic shuttle-box by Reynolds and Casterlin (1976) and Reynolds (1977), in which two environmental variables could be manipulated simultaneously. While four-chamber dynamic shuttle-boxes are presumably complicated to construct as this has not been attempted since the 1970s, the technological development since this time may make constructing such a shuttle-box a more feasible task. Another example is the three-chamber dynamic shuttle-box by Schurmann and Christiansen (1994), where the system temperature would be held stable if the fish occupied the intermediate chamber. A third choice chamber where the ambient environment will be held stable in an otherwise dynamic shuttle-box may be useful when studying sedentary animals that are less willing to shuttle to find a preferred environment (Schurmann and Christiansen 1994). However, if a vital point of the study is to compare relative occupation time in the different choice chambers, for instance, to calculate either avoidance level or to establish that movement has not been random (Schurmann and Steffensen, 1992; Christensen and Grosell, 2018), an intermediate choice chamber represents an undesired occupation space and should not be applied. Furthermore, the intermediate choice chambers may act as a refuge for the animal if it does not exactly resemble that of the other two choice chambers, which introduces an unintended chamber preference bias (Bevelhimer, 1996; Myrick *et al.*, 2004, Reiser *et al.*, 2013).

**Figure 3:**
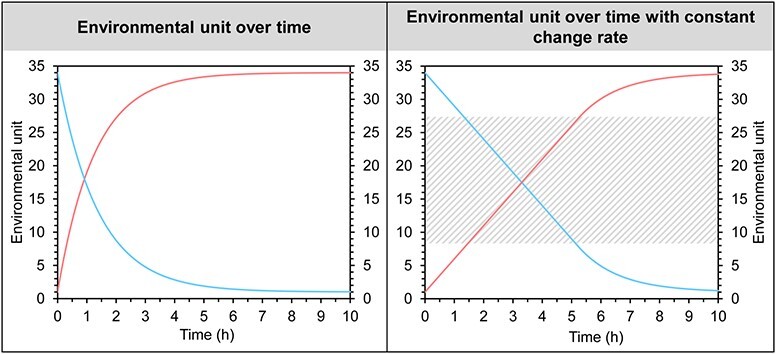
Regulation of any given environmental unit over time in a shuttle-box. The left panel shows the up-regulation of the environmental variable (E) (red line) as E (h) = 33 × (1—e ^−0.723 × h^) + 1 and down-regulation of the environmental variable (blue curve) as E (h) = 33 × e ^−0.723 × h^ + 1. The right panel shows a constant change rate of + or −5 units h^−1^ where possible. Dashed grey area indicates the range where constant change can be maintained for both increase and decrease.

### Experimental control and data acquisition

Computerized experimental control and data acquisition have been inherent parts of shuttle-boxes since the introduction by Schurmann *et al.* (1991). Computerization laid the path video recording to track the position of the animal in real time (Schurmann and Christiansen, 1994). Before computerized real-time video tracking, shuttling of an animal was detected with two photocells, with which the interruption sequence of an animal passing could be used to determine the in which choice chamber the animal was present. The use of photocells to detect shuttling has historically required elongated passages between the choice chambers, which can act as a refuge for the experimental animal (Bevelhimer, 1996; Reiser *et al.*, 2013), but newer narrow-beam miniature photocells may circumvent this issue. With video tracking, taking position in the passage between choice chambers can be avoided by keeping the passage short, which provides a significant advantage for experimenting. Furthermore, having an elongated passage will create areas around the central part of the shuttle-box, which cannot be video tracked (see Figure S2 in the supplementary material). An additional benefit of video tracking is that animal activity within each choice chamber can be recorded as their actual movement distance, and not only as passes between choice tanks.

### Environmental manipulation

#### Regulation

The environmental variable in shuttle-box systems is regulated using an up-and-down, binary approach. For example, to control salinity, the addition of saline or fresh water is in either an on or off state. Up-and-down regulation inevitably has capacity limits and the environmental variable will be logistically approaching respective asymptotes (Fig. 3). In many studies, the environmental variable’s change rate is controlled to be fixed at a certain level. Constant change rates will, however, only be possible if the instantaneous slope of the logistically changing environmental variable is higher than the desired constant change rate (Fig. 3). Beyond the environmental level where the instantaneous slope of the logistically changing environmental variable is lower than the desired constant change rate, the change rate will decrease approaching the capacity limit. Consequently, constant change rates for both increases and decreases of an environmental variable can only be achieved within a certain range (Fig. 3).

Regulation of the environmental variable is generally achieved either by internal treatment of the same body of water (e.g. heating/cooling) or by means of adding water from external reservoirs to conduct gradual water exchange through designated overflows (e.g. adding saline or fresh water). Regulation on the same body of water has the advantage of low water usage, while regulation by adding water can maintain proper water quality over large periods of time (Gregory and Anderson, 1984). Regulation by adding water may be favourable for long-term experiments, as it will avoid build-up of waste products and counter potential leakage of the system (Mortensen *et al.*, 2020). Furthermore, regulation by adding water can also minimize salinity changes due to evaporation, which is especially important in seawater experiments. However, as experiments are usually conducted on fasted animals, regulation on the same body of water is usually sufficient and water exchange can be done between experimental trials (Nay *et al.*, 2020), which will reduce water consumption. An intermediate solution can be to continuously add small amounts of new water to a system that otherwise regulates the environmental variable on the same body of water (Mortensen *et al.*, 2020). However, adding water to a system that also regulates the environmental variable internally should be carefully considered, as it may limit the system’s capacity to dynamically regulate the environmental parameter if not properly adjusted and accounted for (see ‘Dimensioning’ section for more detail). It should be noted that, to date, no salinity shuttle-box exists where the same body of water can be treated and addition of water is therefore an inherent part of this system.

#### Control

**Figure 4:**
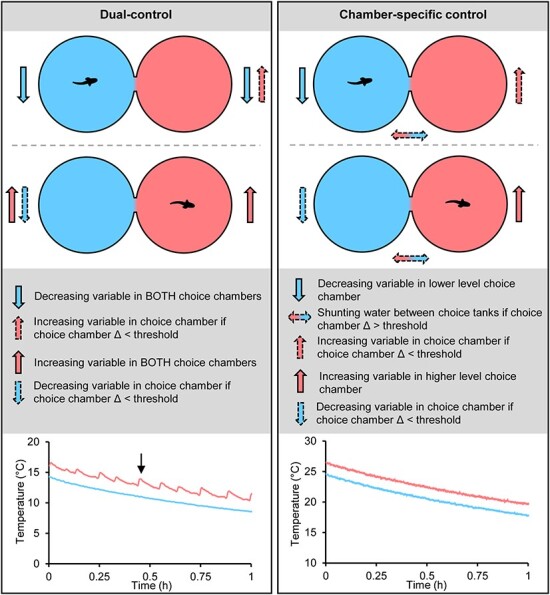
Controlling the environmental variable in shuttle-boxes by means of ‘dual control’ and ‘chamber-specific control’. The data in the graphs originates from Christensen et al. (2021) for the dual-control example and from Christensen et al. (2020) for the chamber-specific control. The black arrow indicates small curve spikes caused by counter-regulation.

There are two distinctly different ways of controlling the environmental variable in modern shuttle-boxes. One way is that the environmental variable can be decreased and increased in *both* choice chambers (e.g. in Frank, 1971; Neill *et al.*, 1972; Stol *et al.*, 2013; Borowiec *et al.*, 2018; Christensen and Grosell, 2018; Cooper *et al.*, 2018; Fig. 4). This methodology will hereby be termed ‘dual control’. Notably, with dual-control shuttle-boxes, the environmental variable will change faster in the choice chamber where the animal is not present than in the choice chamber where it is present due to the environmental difference between the choice chambers combined with the changing environmental variable logistically approaching its respective regulation capacity asymptotes. The environmental variable in the chamber where the animal is not present may therefore, once in a while, require counter-regulation, that is, be increased if the animal is in the lower level choice chamber and be decreased if the animal is in the higher level choice chamber (small curve spikes indicated by black arrow in Fig. 4). This counter-regulation will limit overall regulation capacity and cause the environmental difference between the chambers to vary over time. Excessive counter-regulation in systems using dual-control can possibly be avoided by carefully adjusting hysteresis and flow rates of the system, though this may be a complex and time-consuming process.

Another way of controlling the environmental variable in shuttle-boxes is to *only* increase the environmental variable in the higher-level choice chamber and *only* decrease the environmental variable in the lower-level choice chamber. The environmental difference between the choice chambers is then maintained by shunting water between the choice chambers when the environmental difference between the choice chambers exceeds the desired level (Fig. 4) (e.g. Schurmann *et al.*, 1991; Petersen and Steffensen, 2003; Nay *et al.*, 2015; Christensen *et al.*, 2020). This methodology will be referred to as ‘chamber-specific control’. When using chamber-specific control, counter-regulation will only be necessary if an animal stops shuttling between the choice chambers for extended periods of time. In such cases, the environmental variable may reach the capacity limit, or set minimum or maximum limits, of the system, and the environmental difference between choice chambers decrease due to inevitable exchange of water between choice chambers. Counter-regulation is thus a rare issue in shuttle-boxes using chamber-specific control, resulting in enhanced overall regulation capacity and greater stability in chamber differences over time compared to systems using a dual-control approach.

#### Temperature

The functional use of temperature shuttle-boxes have varied considerably. For instance, the constant temperature difference between choice chambers reported in dynamic temperature shuttle-boxes range between 0.5°C and 4°C, with a median of 2°C. Unfortunately, only a few studies have provided details on their choice of temperature difference between choice chambers, making it difficult to provide any overall best practice recommendation on temperature difference between choice chambers. Of the studies that do give information on the matter is, for instance, Nay *et al.* (2015), who state that a 1°C difference sufficient to make the fish thermoregulate in their dynamic temperature shuttle-box. Interestingly, Neill *et al.* (1972) showed that bluegill sunfish (*Lepomis macrochirus*) thermoregulated behaviourally without a temperature difference, presumably as they learned to associate spatial movement with eventual (but not immediate) temperature change. However, Neill and Magnuson (1974) deemed that a 2°C difference prompted a more precise behavioural thermoregulation than a 1°C difference. While no study mentions it, one could expect that too large of a temperature difference may discourage the animal from moving between chambers. Common for the few studies actually arguing for their temperature difference between the choice chambers is that they do not support their choice with actual data. A quantitative study showing the effect of different chamber temperatures on temperature preference and avoidance of aquatic animals could be a valuable contribution to the literature.

Similarly to temperature difference, the temperature change rate in dynamic shuttle-boxes have also varied considerably and ranges from 1°C h^−1^ to 30°C h^−1^, with the median being 4°C h^−1^. Although no study explicitely addresses it, the temperature change rate should naturally be adjusted to the study animal in question. For instance, slow-moving species (e.g. snails) may need low change rates to enable the animals to shuttle before temperature becomes adverse (Myrick *et al.*, 2004; Reiser *et al.*, 2013). Contrarily, highly active animals, like a pelagic fish, may need a high temperature change rate to keep up with their natural pace of random movement. To our knowledge, no study has attempted to estimate the effect of temperature change rate on the results of dynamic temperature shuttle-box experiments, which should be a target for future research to provide recommendations.

An additional concern to consider in relation to temperature change rate is that water cooling capacity may constitute a limitation for shuttle-box experiments (Stol *et al.* 2013). We have created an adjustable model to calculate the theoretical heating and cooling power needed for a system with a given volume (supplementary ‘Heating-cooling power model’). According to this model, a shuttle-box (32.2 l) with a temperature change rate of 10°C h^-1^ between 15°C and 25°C demands 375 W of heating/cooling power. A larger shuttle-box of 1.5 x the dimensions has 3.4 times as much water (108.7 l) and demands 1264 W of heating/cooling power with the same temperature change rate and within the same temperature range. While a heating of this larger shuttle-box could simply be achieved with more rod heaters, few commercially available single phase chillers are able to cool with this effect. Therefore, a suggestion for standardizing temperature change rates could be to keep it below 10°C h^−1^ to enable comparability among studies conducted in differently sized shuttle-boxes.

**Figure 5:**
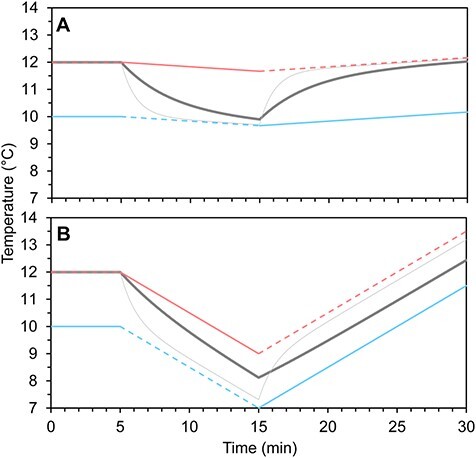
Water temperature and body core temperature of animals in a dynamic temperature shuttle-box. (**A**) shows a modelled temperature change rate of 2°C h^−1^, while (**B**) shows a modelled change rate of 18°C h^−1^. Water temperature in the ‘increasing’ choice chamber (red line) and the ‘decreasing’ choice chamber (blue line) is set with a 2°C difference. Presence in choice chambers is represented by dashed lines, and absence is represented by full lines. The estimated body core temperature is given for a 10-g (thin light grey line) and a 100-g (thick dark grey line) sea raven (*Hemitripterus americanus*) at 1 Hz, based on the mass specific temperature change rate from Stevens and Sutterlin (1976).

It should be noted that animals presumably choose ambient temperatures based on their body core temperature, which in heterothermal environments is dependent on the animal’s recent thermal history. In their experiments using dynamic temperature shuttle-boxes, Reynolds *et al.* (1976) and McCauley *et al.* (1977) equipped fish with stomach thermometers and concluded that there were no differences between ambient temperature and body core temperature. However, the heat transfer rate between ambient water and body core of an animal is largely size dependent (Stevens and Fry, 1974; Stevens and Sutterlin, 1976). In dynamic shuttle-box experiments, body core temperature of animals can be estimated as follows:

$$ \begin{align*} {\mathrm{T}}_{\mathrm{b}}={\mathrm{T}}_{\mathrm{a}}+\big({\mathrm{T}}_{\mathrm{i}}-
{\mathrm{T}}_{\mathrm{a}}\big)\times {e}^{-\mathrm{kt}}, \end{align*}$$

where T_b_ is the body temperature, T_a_ is the ambient temperature, T_i_ is the initial body temperature, k is the change rate of body core temperature and t is the time (min) (Schurmann *et al.*, 1991). The mass specificity of k is species specific and usually expressed as follows:

$$ \begin{align*} \mathrm{k}=\mathrm{a}\times {{\mathrm{m}}_{\mathrm{b}}}^{\mathrm{b}}, \end{align*}$$

where a and b are constants and m_b_ is body mass (g) (Stevens and Fry, 1974; Stevens and Sutterlin, 1976). Examples of modelled body core temperatures of differently sized fish in systems with different temperature change rates are shown in Fig. 5. In these modelled examples, the body core temperature of the 10-g fish reaches equality with the ambient water over time at a change rate of 2°C h^−1^, while there are steady state differences between body core temperature and ambient temperature in the larger fish and with higher temperature change rates. Not accounting for the effect of animal size on body core temperature may therefore cause serious errors in estimates of preference and avoidance temperatures.

#### Carbon dioxide

All CO_2_ shuttle-box studies we found in the literature search have been conducted as avoidance experiments in static shuttle-boxes and have progressively increased CO_2_ level in one choice chamber, while keeping normocapnia in the other. Unfortunately, none of CO_2_ studies found in the data base reports details on the rate by which CO_2_ changes, despite this factor potentially affecting results and study repeatability.

#### Oxygen

For the dynamic O_2_ shuttle-box described by Borowiec *et al.* (2018), the authors have not stated change rate in O_2_ level, yet the constant difference in O_2_ level between the choice chambers was held at 30% dissolved oxygen (DO). All the static O_2_ shuttle-boxes have assessed avoidance O_2_ level by having progressive hypoxia in one chamber, with change rates between 41% and 102% DO h^−1^ (Nati *et al.,* 2018; Ern and Esbaugh, 2021).

#### Salinity

The dynamic salinity shuttle-box studies in the data base have reported using a constant salinity difference between the choice chambers of 3–5 (please note that the SI unit for salinity uses the PSU scale which is by definition dimensionless), but without providing any reasoning as to why. With the existing literature on the salinity sensing in aquatic animals is currently sparse (Kültz, 2012), and static salinity shuttle-boxes having only assessed environmental preference for freshwater or sea water it is therefore difficult to provide any recommendations on the salinity difference between choice chambers necessary to prompt a shuttling response. The effect of salinity difference between choice chambers on salinity preference and avoidance could therefore be target for future studies, which would add to the literature on salinity sensing in aquatic animals.

The salinity change rate has not been specified precisely, but is given as flow rates in Christensen and Grosell (2018), who explicitly state that the system was used without a linear change rate and the system salinity in turn changed logistically towards the asymptotically increasing and decreasing limits.

As both temperature and salinity may interact on the physiology of aquatic animals (Christensen *et al.*, 2017), temperature of the water added to salinity shuttle-boxes must be closely regulated not to add temperature as a confound for salinity preference and avoidance (Christensen and Grosell, 2018).

It should be noted, that salinity shuttle-box systems may require a substantial amount of water. For instance, Christensen and Grosell (2018) estimated a use of up to 1200 L to regulate salinity in their 50 L shuttle-box system over 20 h. Furthermore, reaching fresh water or sea water levels in dynamic salinity shuttle-box systems is somewhat problematic, as these salinities constitute the asymptotic regulation capacity limits and may take very long time to reach.

### Dimensioning

**Figure 6:**
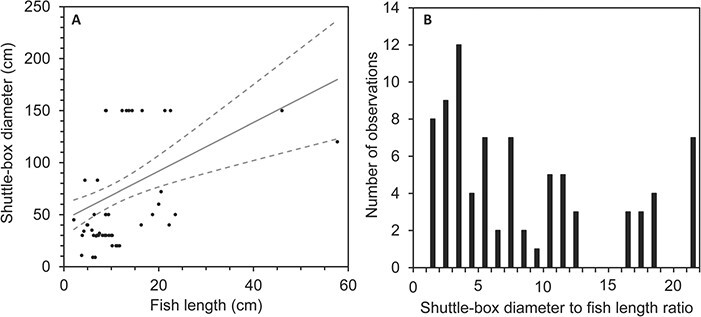
Animal length in relation to choice chamber width (diameter for circular setups). The full line in (**A**) shows a linear regression on animal length (L_a_) and choice chamber width (W_cc_) (W_cc_ = 2.291 × L_a_ + 46.68) and dashed lines represent its upper and lower 95% confidence limits. (**B**) shows a histogram on choice chamber width to animal length ratios. Data are from studies found in the literature review and represented by treatment group as some studies used differently sized systems for different treatment groups. Cooper et al. (2018) repeated experiments in static and dynamic shuttle-boxes and these are therefore compiled.

The size of a shuttle-box will limit the size of animals that can be used in the system. While the choice tanks should be large enough for the animal to move freely and limit confinement stress, a system that is too large will require excessive mixing to homogenize water in each chamber, potentially causing animals to move against a current and experience some degree of stress or exhaustion (Ern and Esbaugh, 2021). To our knowledge, no study has systematically assessed the effects of choice chamber width to animal length ratio, which would be valuable knowledge for planning future studies. Among existing shuttle-box studies, animal size ranged from 0.5 to 431.8 g and 2.1 to 57.7 cm, covering all life stages from larvae to adults (e.g. Reynolds and Casterlin, 1978b; Christensen and Grosell 2018; Bulkley and Pimentel, 1983), while the width (diameter of circular setups) of the choice chambers varied from 9 to 150 cm. Of the studies in the database that reported both choice chamber width and animal size, we found a significant linear regression relationship between the two parameters (ANOVA, *F* (1,77) = 14.33, *P* = 0.0003). The choice chamber width to fish length ratio in these studies ranged from 1.35 to 21.43, with a mode of 3 (when rounding off to whole numbers). While there is a large variation in choice chamber width to animal size, as evident of the low goodness of fit in the regression analyses (R^2^ = 0.161), the most commonly choice chamber width to fish length ratio used is between 3 and 4, representing the most frequent interval in Fig. 6.

Apart from the size of the animal, a primary concern with dimensioning a shuttle-box is that the capacity range of the environmental variable in question, along with its change rate capacity, depends on the total water volume of the system and the maximum regulation capacity. Water volume and environmental regulation capacity are therefore important to consider for shuttle-box experiments (Stol *et al.*, 2013). Regulation capacity for various environmental variables is elaborated on in the Supplementary material.

### Availability

Shuttle-box systems can be bought commercially or be custom made. The current price for a commercial shuttle-box setup is around €15 000 (www.loligosystems.com, as per February 2020), excluding computer, backlight illumination, and regulation equipment (e.g. heating-rods and chillers, which can add around €3500 or more in additional costs). While custom-made shuttle-boxes may take time to build, they will naturally reduce the costs of acquiring a shuttle-box considerably. There has, to our knowledge, not been made a publicly available freeware for experimental control and data acquisition in shuttle-box experiments. For a custom-made shuttle-box, time may therefore also be needed to code the software for experimental control and data acquisition. The creation of experimental freeware, such as AquaResp® for respirometry studies (www.aquaresp.com), may increase the availability and use of shuttle-box systems and could be a target for future endeavours.

It should be noted that the current commercially available shuttle-box uses the dual-control methodology (Stol *et al.*, 2013), which has a less effecient environmental regulation capacity compared to the chamber-specific control methodology. It is also worth noting that the current commercially available shuttle-box has an elongated passage, which effectively acts as a third chamber where the environmental variable will be held stable if the animal occupies it (Stol *et al.*, 2013; Christensen and Grosell, 2018). Such an elongated passage may act as a refuge and potentially bias the results (Bevelhimer, 1996; Myrick *et al.*, 2004, Reiser *et al.*, 2013) and potentially compromise effective video tracking (see Figure S2 in the Supplementary material).

**Table:** 

Summary box for: ‘shuttle-box systems’	
Element	Synthesis
Choice chambers	Circular choice chambers eliminate corners as hiding places and provides more effective water separation between chambers Short choice chamber passage eliminate passage as hiding place and improves tracking
Mixing chambers	Provide even mixing in choice chamber and enable aeration/gas manipulation without disturbing animal
Environmental control and regulation	‘Chamber-specific control’ methodology most effectively avoids excessive counter-regulation, in turn improving regulation capacity The appropriate choice chamber environmental difference and change rate depend on animal activity and environmental sensing ability
Experimental control	Video tracking enables experimental control and precise activity measurements
Dimensioning	The most common choice chamber width to fish length ratios are between 2 and 4 Water volume will determine the maximum regulation capacity and should be carefully considered
Experimental control	Video tracking for shortening of choice chamber passage and possibility for precise activity measurements
Identified knowledge gaps Development of modern four chamber—two environmental variable shuttle-box Systematic assessment of effect of environmental difference between choice chambers Systematic assessment of effect of environmental change rates in shuttle-boxes Using shuttle-boxes to study salinity sensing in aquatic animals Systematic assessment of proper choice-chamber-diameter-to-fish-length ratios Development of experimental control and data acquisition freeware	

## Shuttle-box experiments

### Experimental design

#### Acclimation history

An animal’s acclimation history prior to experimentation can affect its preferred environmental range. For instance, acclimation temperature has been shown to affect both temperature preference and avoidance in a variety of species (Reynolds and Casterlin, 1979a; Habary et al., 2016; Barker *et al.*, 2018). Furthermore, acclimation temperature has been shown to affect CO_2_ avoidance (Cupp *et al.*, 2017; Tix *et al.*, 2018). Only one study has assessed the effect of CO_2_ acclimation on CO_2_ avoidance and showed that avoidance increases with increasing acclimation level (Dennis *et al.*, 2016). Feeding history can also affect environmental preference. For instance, Bucking *et al.* (2012) showed that diet largely influences salinity preference in killifish (*Fundulus heteroclitus*), while Killen (2014) showed that preferred temperature depended on the feeding history of common minnows (*Phoxinus phoxinus*). However, Suski *et al.* (2019) did not find an effect of nutritional status on CO_2_ avoidance level in largemouth bass (*Micropterus salmoides*). Acclimation history should therefore be carefully planned before experimenting.

A prerequisite of assessing the effect of environmental acclimation on environmental preference and avoidance is that animals have regained homeostasis after environmental change. The time it takes for animals to regain homeostasis after transfer to new environments (e.g. laboratory settings and altered environmental levels) depends on the environmental variable in question and the rate and magnitude of change and the study species, but this process may take several weeks (e.g. Sidell *et al.*, 1973; Morgan and Iwama 1998; Bouchard and Guderley, 2003; Serrano *et al.*, 2011). Of the shuttle-box studies we surveyed, only 59% provided information on acclimation time prior to shuttle-box experiments. Of these, acclimation time prior to experiments ranged from 0 to 28 weeks, with 3 weeks on average (median).

#### Dynamic or static shuttle-box?

While both dynamic and static shuttle-boxes can be used to determine environmental preference and avoidance, they each have their best use in specific contexts. For instance, a stable measurement of an individual animal’s temperature preference can be reached for most species in dynamic temperature shuttle-box within 24 h (Macnaughton *et al.*, 2018; Harman *et al.*, 2020), while it took Larsson (2005) 16 days to determine temperature preference of individual Arctic charr (*Salvelinus alpinus*) in a static shuttle-box through a series of paired temperatures choice tests. The dynamic shuttle-box is thus probably best fit to determine environmental preference. However, environmental avoidance in dynamic shuttle-boxes may be affected by the animal learning how the system works and keeping it more precisely at a steady state around preferred environmental level (Clingerman *et al.*, 2007). Therefore, static shuttle-boxes are probably more suitable for determining environmental avoidance (Cook *et al.*, 2011; Kates *et al.*, 2012; Tucker and Suski, 2019). To our knowledge, no study has compared environmental avoidance determined in dynamic and static shuttle-boxes, which could be a valuable target of future studies. To date, only static shuttle-boxes have been used to study effects of additional biotic factors, such as sociability or predator presence, on preferred environmental ranges (Tietze and Gerald, 2016; Cooper *et al.*, 2018; Tucker and Suski, 2019).

#### Pre-disposed choice chamber preference

Differences in ambient settings, e.g. unevenly distributed light, can potentially influence the spatial occupation in setups for determination of environmental preference and avoidance (Scherer and McNicol, 1998) and is therefore an issue that should be dealt with ahead of experimentations. Choice chamber difference can, to a large extent, be minimized by keeping the ambient conditions in shuttle-boxes (e.g. choice chamber colour, choice chamber dimensions, light level, etc.) as even as possible between the two choice chambers. In shuttle-boxes, potential pre-disposed choice chamber preference can be assessed in a sub-study/pilot study, as in Tattersall *et al.* (2012), or accounted for experimentally and statistically by switching the orientation of the high/increasing environmental level and the low/decreasing environmental level choice chambers between trials (Christensen and Grosell, 2018; Nati *et al.*, 2018; Harman *et al.*, 2020). Alternating between which choice chamber animals are placed at the beginning of experiments has also been done (Stol *et al*., 2013). However, this approach will not address any potential chamber bias due to an actual ambient condition, e.g. light or colour differences, other than the animal perceiving the choice chamber it is first placed in as different from the other. It should be noted that pre-disposed choice chamber preference has been used to prime a precise avoidance reaction in experiments, and can thus be used as an advantage (Cook *et al.*, 2011; Herbert *et al.*, 2012; Mucha *et al.*, 2020).

### Experimental duration

#### Initial system acclimation

During behavioural experiments it is common for animals to become hyperactive or show freezing behaviour and stay immobile after transfer to an experimental arena (e.g. Maximino *et al.*, 2010; Tattersall *et al*., 2012). Consequently, initial behaviour in shuttle-box studies may be due to anxiety or stress, rather than environmental preference or avoidance. An initial period with a static environmental setting may therefore be beneficial for allowing the animal to calm after handling and settle in the system (i.e. an initial system acclimation period). Among the studies in our literature review, the initial system acclimation period that was used varied from 0 h (acutely started trials) to 10 days, with a median time of 1 h. However, justification for the length of initial system acclimation period is often lacking. While some studies report that their initial system acclimation period was enough for animals to stop being ‘explorative’ (Suski *et al.*, 2019; Tucker and Suski, 2019; Tucker *et al.*, 2019), we found only one study that had systematically assess the need and length of initial system acclimation period: Harman *et al.* (2020) showed that initial settling period in a dynamic shuttle-box did not affect the resulting temperature preference in lake whitefish (*Coregonus clupeaformis*) yet affected the variation among individuals. The length of initial system acclimation period in shuttle-boxes may be largely system (dynamic/static, environmental variable) and species depend, but Harman *et al.* (2020) present a useful framework for assessing this issue in future studies.

#### Duration of dynamic shuttle-box trials

The total duration of a dynamic shuttle-box trial should be long enough to elicit a consistent pattern in the animals’ choice of environment. This will inevitably depend on the time it takes for system to gravitate towards a stable level around an animal’s preference (gravitation time). Gravitation time will primarily depend on the environmental change rate of the system and the difference between the initial environmental level and the preferred level of the animal. For instance, in a trial where the initial temperature is 10°C, the temperature changes by 5°C h^−1^, and the animal’s temperature preference of 20°C, the minimum gravitation time will be 2 h. However, if the initial temperature was 20°C, the minimum gravitation time would be 0 h. Secondly, gravitation time may depend on a species ability to learn how to change the environment in the system. It is possible that having an initial system acclimation period with a static environmental difference between the choice chambers may facilitate learning of the environmental differences between the chambers. Such predisposed understanding of the environmental difference between the choice chambers may facilitate a faster understanding of the connection between the environmental difference between the choice chambers and the change in the overall level of the environmental variable, and thus reduce gravitaion time.

A stable measurement of preference in dynamic shuttle-box studies has, in many cases, been shown to occur within 24 h for both temperature and salinity (Bucking *et al.*, 2012; Killen, 2014; Habary *et al.*, 2016; Macnaughton *et al.*, 2018; Harman *et al.*, 2020). For O_2_, trial length has not been reported (Borowiec *et al.*, 2018), and for CO_2_, no dynamic shuttle-box experiment has, to our knowledge, been used in any published study. A few authors have noted that gravitation time lasts only a few hours (Reynolds, 1977; Harman *et al.*, 2020; Christensen *et al*., 2021), while it took ~10 h in Siikavuopio *et al.* (2014). Note that we have included a suggestion for how to statistically calculate and assess gravitation time in the ‘Data analyses’ section later in the present review.

**Figure 7:**
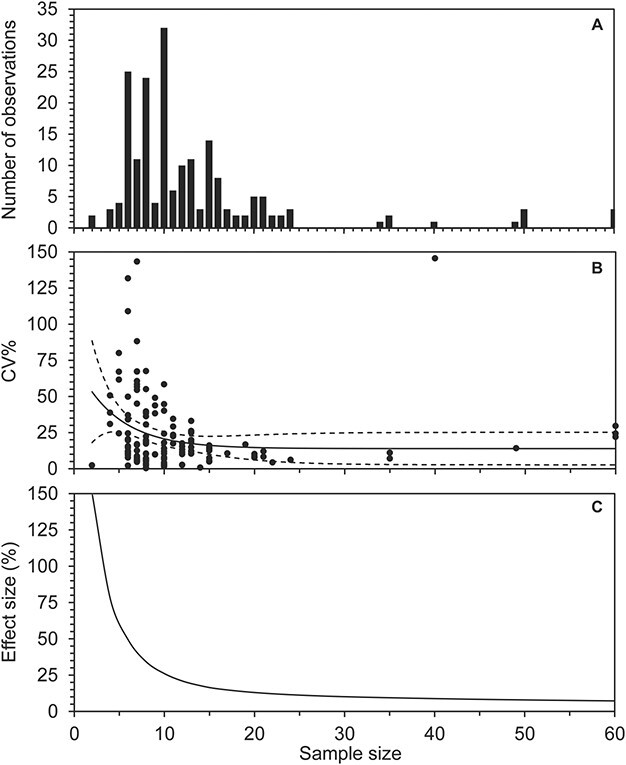
Sample size used for shuttle-box studies and their general variance and statistical power. In (**A**), the used samples sized are sorted in a histogram. (**B**) shows the coefficient of variation (CV%) in relation to sample size and a fitted asymptotic exponential decay function (full line). Dashed lines represents 95% confidence limits. (**C**) shows the effect size between the mean of two treatments that can be statistically differentiated with the CV% of the corresponding sample size.

#### Duration of static shuttle-box trials

There are in essence two ways of using static shuttle-boxes, and each will determine the duration of experimental trial by different means. The first one is to measure occupation time in two compartments with different levels of an environmental variable (e.g. Larsson, 2005; Tietze and Gerald, 2016; Nati *et al.*, 2018). Here, animals are given a fixed amount of time to choose between a set of static environmental conditions, and most studies repeated this procedure over a gradient of paired environmental levels. Duration of each period with a paired set of environmental conditions have mostly been between 10 min and 8 h (e.g. Nielsen and McGaw, 2016; Tietze and Gerald, 2016), but also lasted 2–3 days (Larsson, 2005; Norin *et al.*, 2014) (excluding initial system acclimation periods). The second principal way of using static shuttle-boxes is to progressively increase the environmental variable in the choice chamber where the animal is present until the animal chooses to escape into the alternative choice chamber (e.g. Frank, 1971; Cook *et al.*, 2011; Herbert *et al.*, 2012; Kates *et al.*, 2012). The avoidance part of these experiments is usually over within a few hours. If initial system acclimation periods are carried out overnight, it seems that the most avoidance trials in static shuttle-boxes can be conducted within the duration of a working day.

### Sample size

The sample size that has been used in shuttle-box studies ranges from 2 to 60 with a median value of 10, based on all treatment groups and all metrics (preference, lower avoidance and upper avoidance levels) in the database for both dynamic and static shuttle-box studies (Fig. 7A). To estimate the variation in relation to sample size, we fitted an asymptotic exponential decay function of coefficient of variation (CV) in relation to sample size of the studies in the database (Fig. 7B). The fit estimated that the level where an increase in sample size no longer reduces the measured variation is 14% (the asymptotic level). A sample size of 7 has a corresponding CV of 28%, that is, double as high as the asymptotic level. In comparison, a sample size of 20 (double the most commonly used sample size in shuttle-box studies) yields a CV of 15%, and thus much closer to the asymptotic CV% level. Samples sizes towards 20 will thus likely provide a precise measure of the variation in an environmental preference determined in a shuttle-box study. However, if conducting a comparative study sample size in combination with the CV will determine the effect size that can be distinguished statistically. We used the CV vs. sample size fit to perform power analyses to determine the effect size (%) that can be statistically differentiated with a significance level of 0.05 and a power of 0.8 in relation to sample size (Cohen, 1988; Fig. 7 C). The power analysis showed that the general detectable effect size for a sample size of 7 per treatment group is 40%, while it was 13% for a sample size of 20 per treatment group. The CV may naturally vary with a variety of factors, such as the environmental variable in question, the methodological approach and species phylogeny which might explain some of the residual variation in the CV vs. sample size fit (R2 = 0.1569). While we did not analyse cofactors in the present study, any methodological optimization may move the CV and effect size curves in Fig. 7 down towards the x-axis.

### Experimental throughput

Considering that the standard of shuttle-box trial duration is 24 h and a reasonable sample size may approach 20 per treatment group, experimental throughput can potentially be an issue in shuttle-box studies. Although the repeatability of preference may be high within a week (Killen, 2014), temperature preference has been shown to change with ontogenetic life stage/body size in many studies (Medvick and Miller, 1979; Burleson *et al.*, 2001; Lafrance *et al.*, 2005; Larsson, 2005; Cooper *et al.*, 2018; Christensen *et al.*, 2020). Significant changes in body mass over time, e.g. due to growth or due to wild animals refusing to eat in laboratory settings, may therefore cause significant variation in environmental preference, and further lower statistical power. In turn, low experimental throughput may limit studies on ontogeny of one cohort, especially in smaller individuals/younger life stages and in warm conditions where somatic turnover of ectotherms is more rapid.

**Table:** 

Summary box for: ‘shuttle-box experiments’	
Element	Synthesis
Acclimation time	A total of 3 weeks in most instances
Dynamic or static shuttle-box?	Dynamic for preference Static for avoidance
Pre-disposed choice chamber preference	Should be accounted for experimentally
Experimental duration	Initial system acclimation times are beneficial Most studies can be conducted within 24 h
Sample size	Between 7 and 20
Experimental throughput	Constitutes a severe limitation in shuttle-box studies
Identified knowledge gaps Comparison of avoidance levels in static and dynamic studies Development of high-throughput, low-cost system	

Harman *et al.* (2020) provides an excellent example of the relationship between experimental throughput and statistical power. With power analyses, Harman *et al.* (2020) estimated that having a daily throughput with three trials per day using a trial lenght of 4 h the same statistical power (0.6) was reached in 32 days compared to 45 days when using a daily throughput of one with a trial lenght of 24 h. However, an experimental trial of 4 hours and a daily throughput of three would demand workdays of more than 12 h in the laboratory, if the task is not split up between multiple people, which is not compatible with a healthy work–life balance in the long run (Kinman and Jones, 2008). Alternatively, having multiple setups could also be used to increase experimental throughput, whereby longer trials yielding more precise data could be run by a single experimenter. Surprisingly, we have found only one study that utilized multiple setups in our literature search (Neill and Magnuson, 1974), probably owing to the space that multiple shuttle-boxes would demand. Furthermore, the cost of acquiring commercial shuttle-boxes may be a severe limitation to having multiple systems. To solve the problem related to the cost of multiple setups, development of an low-cost, high-throughput system consisting of multiple shuttle-boxes, such as the system recently described for respirometry studies (Drown *et al.*, 2020), would be a welcomed endeavour for future studies. For this, the detailed description of how to build and set up a custom-made shuttle-box provided in the supplementary material could be a starting point.

## Data analysis

Commonly for all shuttle-box study, initial system acclimation period, where the system is held statically for the animal to accommodate to the shuttle-box system, should naturally not be considered in the calculation of environmental preference and avoidance. Note that some individuals may not explore and regulate during a substantial part of the trial duration, or even not start to regulate at all (Reynolds and Casterlin, 1979b; Enders *et al.*, 2019; Skandalis *et al.*, 2020). If the time spent constitutes a substantial part of the experimental trial, it may affect the subsequent analyses. Animals that are not regulating may be excluded from analyses (Reynolds and Casterlin, 1979b; Enders *et al.*, 2019; Skandalis *et al.*, 2020), which probably happens more often than is being reported. However, exclusions of individuals may remove valuable information, especially in studies of individual variation and trait correlations. Generally, exclusion of individuals needs to be based on rigorous criteria, which should be assessed and determined systematically. Such an analysis is yet to be performed and published.

### Dynamic shuttle-box studies

**Figure 8:**
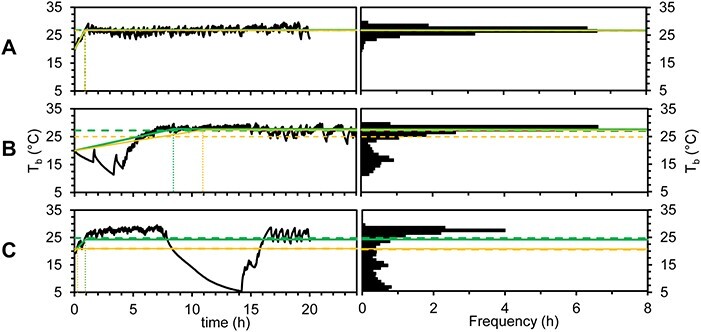
Body temperatures (T_b_; black lines and bars) of individual *Perca fluviatilis* over 20–24 h in a dynamic shuttle-box. Overall mean (orange dashed line), overall median (green dashed line), temperature preference (T_pref_) determined with a parametric two line segmental linear regression (orange full line) and T_pref_ determined with a robust two line segmental linear regression (green full line) are shown. The gravitation time (t_g_) is shown as vertical dashed lines. The fish in (**A**) had a short t_i/g_ and thermoregulated within a narrow range of T_b_ that was approximately normally distributed, and all T_b_ estimates are therefore similar. In the example in (**B**), the fish had a longer t_i/g_ where it occupied colder temperature, and the T_b_ frequency distribution is therefore skewed, affecting the overall mean markedly. In (**C**), the fish has a short t_i/g_, but stops thermoregulating for an extended period in the middle of the experiment, in which instance the overall median and robust segmental linear regression best represent the T_pref_ of when the fish actually thermoregulates. It can also be seen from (B) and (C) that the robust segmental linear regression most accurately determines the point where T_b_ stabilizes during experiment (t_i/g_) when T_b_ shows a skewed distribution.

In dynamic shuttle-box studies, environmental preference is typically described by the central tendency (mode, mean, median) of the environmental levels selected by an animal during a trial. Modal selected environmental level most precisely resembles the definition of environmental preference, that is, the environmental level that is most frequently occupied in a free choice situation (Reynolds and Casterlin, 1979a). However, if an animal has frequently selected a broad range of environmental levels during a trial the mode may not be distinct and can be largely affected by altering the resolution of which environmental levels are assessed (typically the bin range of a histogram; Fig. 8; Schurmann *et al.*, 1991).

Compared to mode, the mean is less sensitive to the variation around the central tendency. Note that the mean incorporates unusual and extreme events, such as temporarily choosing another environment that the ‘norm’. These ‘unusual’ events may be of significance, e.g. changes between nighttime and daytime environmental preference (e.g. Serrano *et al*., 2010) or feeding (Reynolds and Casterlin 1979a). However, the ‘unusual’ events may also be caused by random effects, e.g. if the animal is using a long period of time at environmental levels well away from its overall norm in the beginning of the experiment (Fig. 8B), or if the animal temporarily stops shuttling and thus does not actively choose its ambient environment (Fig. 8C). Using mean as a measure for environmental preference should therefore be used with caution.

The median is less affected by unusual events during a shuttle-box trial than the mean (Schurmann *et al.*, 1991). Furthermore, mean and median are the same if the variation is evenly distributed around the central tendency. Therefore, if the animals within a study show selected environmental levels sometimes with skewed distributions, sometimes with normal distributions and sometimes with broad ranges of frequently occupied environmental levels, the median can be a robust measure of environmental preference (Schurmann *et al.*, 1991). Notably, if the variation around the central tendency is of interest, for instance to study within- or among-individual variation, it may be valuable to determine range, standard deviation and skewness (Casterlin and Reynolds, 1979; Reynolds and Casterlin, 1979b).

**Table:** 

Summary box for ‘data analysis’	
Element	Synthesis
All studies	Initial acclimation time should be excluded from data analyses Individuals not regulating can be excluded from analyses, according to strict criteria
Dynamic shuttle-boxes	Preference can be determined as overall mode, mean or median Median is recommended if individuals show different distributions of inhabited environmental levels Gravitational time should be accounted for. A two-line segmental linear regression can be used to simultaneously determine gravitational time and preference level Avoidance can be determined by total occupied range, average turnaround levels or quartiles
Static shuttle-boxes	
Identified knowledge gaps Quantitative exclusion criteria of individuals Comparison of avoidance levels in static and dynamic studies	

**Figure 9:**
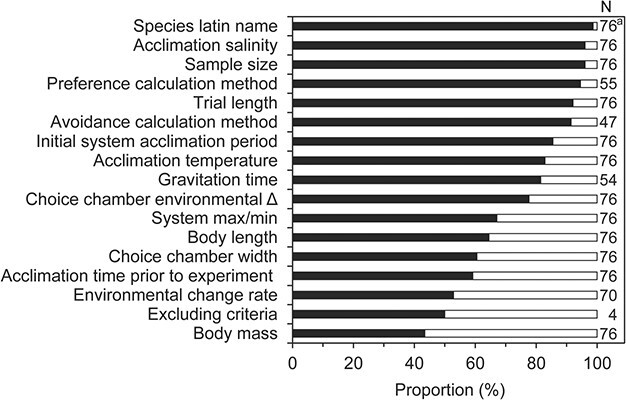
The relative proportion of reporting (black bars) and non-reporting (open bars) of important factors in shuttle-box experiments. ^a^Barker et al. (2018) only reported genus.

Gravitation time has often been excluded from data analyses (e.g. (Casterlin and Reynolds, 1979; Killen, 2014; Habary *et al.*, 2016; Christensen and Grosell, 2018), and the length of the gravitation time is often loosely defined. Although Macnaughton *et al.* (2018) found that environmental preference was dependent on trial length and not gravitation time, this is clearly not the case in Siikavuopio *et al.* (2014). A suggestion for how to simultaneously determine gravitation time and take it into account for the determination of environmental preference (E_pref_) is to fit a two-line segmental linear regression to the occupied environmental levels (E_o_) over time (t) (Christensen *et al.*, 2021):

$$ \begin{align*} {\mathrm{E}}_{\mathrm{o}}{(t)}_1&=\mathrm{a}\times \mathrm{t}+{\mathrm{E}}_{\mathrm{accl}},\\ {\mathrm{E}}_{\mathrm{o}}{(t)}_2&={\mathrm{E}}_{\mathrm{pref}}, \end{align*}$$

where the intercept of the initial segment (E_o_(*t*)_1_) is the acclimation environmental level (E_accl_) while the subsequent segment (E_o_(*t*)_2_) is slopeless ((a) is 0) and its intercept is regarded E_pref_ (Fig. 8). The intersection between the two segments is then a measure of the gravitation time (t_g_):

$$ \begin{align*} {\displaystyle \begin{array}{l}\mathrm{a}\times {\mathrm{t}}_{\mathrm{g}}+{\mathrm{E}}_{\mathrm{accl}}={\mathrm{E}}_{\mathrm{pref}}.\\ {}\kern3.25em \updownarrow \\ {}{\mathrm{t}}_{\mathrm{g}}=\Big({\mathrm{E}}_{\mathrm{pref}}-{\mathrm{E}}_{\mathrm{accl}}\Big)/\mathrm{a}.\end{array}} \end{align*}$$

Such analysis should preferably be robust/non-parametric (green full lines in Fig. 8) to minimise the effects of skewed distributions of occupied environmental levels. Note that some individuals may have longer periods of time during an experimental trial where they do not shuttle between the choice chambers to regulate their ambient environments (e.g. Fig. 8C, from hour 8 to hour 14). Shorter periods with no regulation of ambient environment can, to a large extent, be accounted for analytically by using overall median or robust segmental linear regression to calculate environmental preference.

Researchers have also defined lower and upper environmental avoidance levels from dynamic shuttle-box experiments. Some studies have simply used the environmental range selected by an animal as expressions of avoidance (e.g. Reynolds and Casterlin, 1977; Bulkley and Pimentel, 1983; Serrano *et al.*, 2010), while others have used percentiles (e.g. 25th and 75th: Medvick and Miller, 1979; and 30th and 70th percentiles: Konecki *et al.*, 1995) or mean/median turnaround environmental levels (Beitinger, 1974; Schurmann *et al.*, 1991; Barker *et al.*, 2018). Which measure to use as avoidance in dynamic shuttle-boxes would benefit from a quantitative comparison with avoidance levels derived from static shuttle-boxes, but such study has, as earlier mentioned, unfortunately never been conducted.

### Static shuttle-box studies

Environmental preference can, by its definition (Reynolds and Casterlin, 1979a), only be determined in static shuttle-boxes through a series of experiments of paired environmental level choice tests covering a significant range of environmental levels (McCauley, 1977; Larsson, 2005). In static shuttle-boxes, environmental preference will be the environmental level that yields precisely 50% occupation in one of the choice chambers, which for instance can be found by logistic regression (Larsson, 2005). In static shuttle-boxes that change the environmental difference between the choice chambers stepwise, environmental avoidance can be assessed as the environmental level that yields a statistical difference in occupation time between choice chambers (Kates *et al.*, 2012, Nati *et al.*, 2018; Ern, 2019). In systems that progressively change the environmental variable in one choice chamber, avoidance can be determined as the level that causes animals to escape from their occupied choice chamber (Cook *et al.*, 2011; Kates *et al.*, 2012).

## Study reporting

From all the parameters noted in the database, we analysed the proportion of studies that reported/did not report said parameter (Fig. 9). As outlined throughout the present review, most of these parameters can influence environmental preference and avoidance in most study designs. Omitting to report these parameters may limit general comparison between studies and experimental repeatability. We therefore encourage researchers to search through the parameters given in Fig. 9 as an inspiration to what information should be included in future studies.

## Future directions

With preferred environmental ranges being behavioural manifestations of animals’ physiological response to their environment, we have probably not seen the last shuttle-box study in climate change contexts. Furthermore, with the current global biodiversity crisis, for instance due to invasive species (Walther *et al.*, 2002), shuttle-boxes studies could prove valuable in providing further knowledge on preferred environmental ranges of invasive species where distribution would potentially need mitigation effort (Barker *et al.*, 2018; Christensen *et al*., 2021), or on species that are of conservation concern (Stol *et al.*, 2013).

Another current research topic where shuttle-box studies could be used is individual variation of physiological and behavioural traits (e.g. Burton *et al.*, 2011; Norin and Malte, 2011; Metcalfe *et al.*, 2016; Norin and Gamperl, 2017). To our knowledge, variation in preferred environmental ranges among individuals and on individuals over time has not been studied much and could be interesting topics to pursue in the future. In that context, repeatability of environmental preference and avoidance over long time spans would be essential to assess, which will be important knowledge for experimental studies on preferred environmental ranges in general.

## Conclusions

The shuttle-box has been a popular methodology for studying preferred environmental ranges of aquatic animals over the past 50 years. The system has been applied to a wide range of research topics with regards to preferred ranges of temperature, CO_2_, salinity and O_2_ in a vast diversity of species. By synthesizing the current state-of-the-art of the methodology, we have provided the best practice guidelines with regards to setup, data analyses, experimental design and study reporting. During this process, we have identified a series of knowledge gaps, which can and should be addressed in future studies. Furthermore, we have highlighted some obvious directions for research within evolutionary biology and behavioural and physiological ecology.

## Funding

E.A.F.C. was supported by the Carlsberg Foundation (grant number CF19-0400). SSK was supported by Natural Environment Research Council (NERC) Standard Grant NE/T008334/1.

## Supplementary Material

suppl_data_coab028
