# Supplementary material for: Shuttle-box systems for studying preferred environmental ranges by aquatic animals
Source: Conserv Physiol. 2021 May 17;9(1):coab028. doi: 10.1093/conphys/coab028 (PMC8129825; doi:10.1093/conphys/coab028)
Supplement: suppl_data_coab028 [file suppl_data_coab028.zip › How to build and set up shuttle-boxes final.docx]

How to build and set up shuttle-boxes

Supplementary material to: Shuttle-box systems for studying preferred environmental ranges by aquatic animals

By

*Emil AF Christensen^a,*^, Lars Emil Juel Andersen^b^, Heidrikur Bergsson^b^, John F Steffensen^b^, Shaun S Killen^a^*

*^a^* *Institute of Biodiversity, Animal Health and Comparative Medicine, University of Glasgow, United Kingdom*

*^b^* *Department of Biology, University of Copenhagen, Denmark*

** Corresponding author: emilflindt@hotmail.com*

# Overview

As shuttle-box experiments involves live animals, they should be placed in secluded and shielded places to minimize observer bias from experimenters and random passersby. We also recommend that all electric parts that are in contact with water should be grounded to prevent electric shocking of the experimenter, especially when conducting experiments in seawater. Electrical equipment that is not water-proof and electric junctions, e.g. plugs, should be kept well away from the shuttle-box set-up, and preferably above water level, to prevent short circuiting in case of water spilling and system leak. Figure S1 shows full schematic overviews of the commercially available dual control shuttle-box and the chamber-specific control shuttle-box originally designed by Shurmann *et al*. (1991) and refined in Petersen and Steffensen (2003).An interactive 3D drawing of a dual control shuttle-box for temperature can also be found at [www.researchgate.net/project/Steffensen-shuttle-box-for-temperature-preference-how-to-build-and-run-it](http://www.researchgate.net/project/Steffensen-shuttle-box-for-temperature-preference-how-to-build-and-run-it).

| 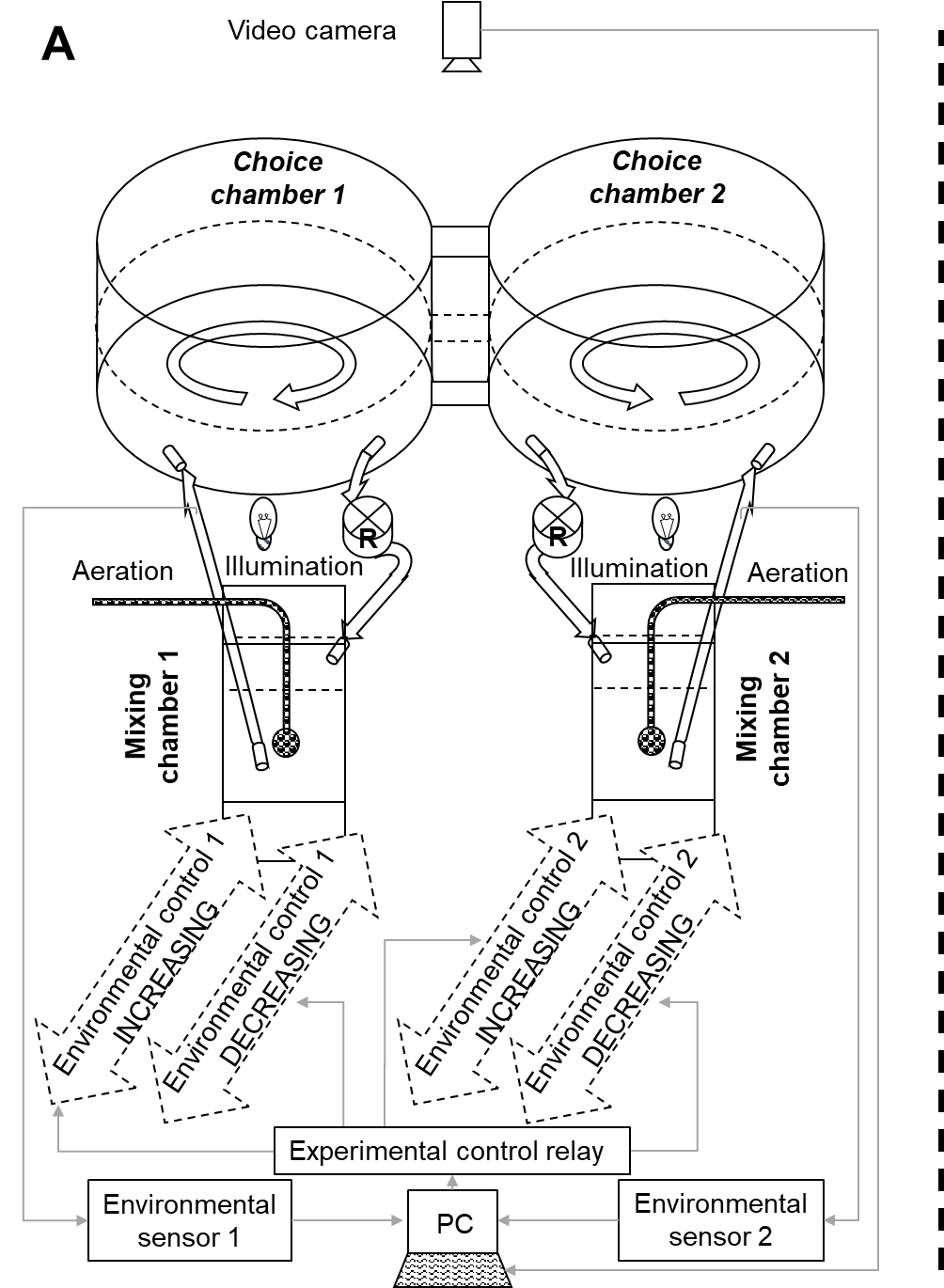 | 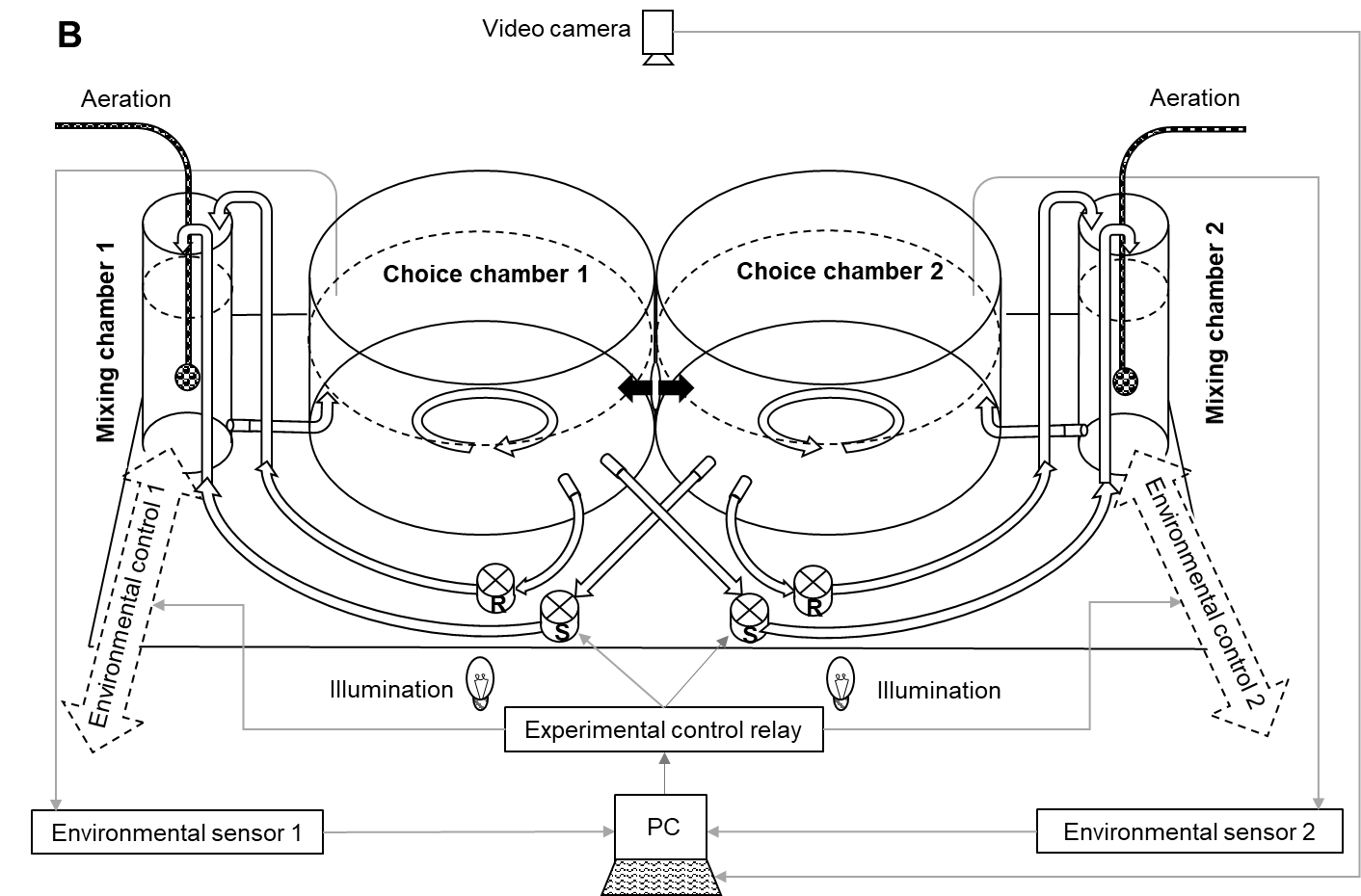 |
| --- | --- |

*Figure S1: Schematic drawing of the commercially available dual-control shuttle-box(panel A) and a chamber-specific shuttle-box (panel B). White arrows symbolizes water movement and direction while dashed lines indicate water level. “R” designates recirculation pumps, “S” designates shunt pumps, black arrow shows the passage between choice chambers, and big dashed double arrows indicate environmental variable control. Grey arrows symbolizes signaling pathways for experimental control and data acquisition.*

# Choice and mixing chambers

Choice and mixing chambers can be bought commercially or be custom made. As starting point for a custom-made shuttle-box, a blue-print of the choice and mixing chambers of a dual control shuttle-box prototype is provided in the supplementary material “Shuttle-box blue-print”. 3D drawing files of the blue print can also be retrieved from [www.researchgate.net/project/Steffensen-shuttle-box-for-temperature-preference-how-to-build-and-run-it](http://www.researchgate.net/project/Steffensen-shuttle-box-for-temperature-preference-how-to-build-and-run-it). Researchers should ensure that materials for 3D printing are water-tight , and for workshop manufacturing, we recommend PVC, as it can be glued and heat-welded. All vertical parts should be either opaque or non-transparent, and the bottom of the choice chambers system is semi-transparent to enable backlight illumination from underneath the set-up. The inlet to the choice chambers should direct the water tangentially into the system and that the water inlets (e.g. with the “diffuser” in the supplementary material “Shuttl-box blue-print”) are oppositely directed to create circular and oppositely directed water currents, and thus a concurrent waterflow along the passage between the choice chambers. Note that certain species with tendencies of leaping might require some refitting, post-commissioning, if the blue print shuttle-box in the supplementary materialsis used, to accommodate the need for a higher barrier. In our experience, preventing escape with netting may affect tracking, and transparent covers tend to get foggy and hinder video tracking. We therefore recommend using tall choice chamber walls to prevent accidental escapes during trials.

Aeration, which should always be provided to prevent undesired hypoxia and hypercapnia, should occur continuously in the mixing chambers to avoid disturbing the experimental animal. The mixing chambers should be tall and slender to enable efficient aeration, or gassing if conducting preference or avoidance experiments in relation to O_2_/CO_2_ levels. Having the water entering the mixing chamber from above with a 180° bend fitting (see on interactive 3D drawing at [www.researchgate.net/project/Steffensen-shuttle-box-for-temperature-preference-how-to-build-and-run-it](http://www.researchgate.net/project/Steffensen-shuttle-box-for-temperature-preference-how-to-build-and-run-it)) and the water outlet low in the water column will prevent issues with air being sucked into the outflowing water: air in the outflow will cause the water level in the mixing chamber to rise until the pressure head is large enough to move the air in the tube, which will cause uneven flow in the choice chambers.

The passage between the choice chambers should be as short as possible to prevent it from becoming an unwanted refuge between the two choice chambers, (Bevelhimer, 1996; Myrick *et al.*, 2004) and to not compromise effective video tracking (see later in present text). The cross-sectional area of the passage between choice chambers should be large enough for the animal to pass but as small as possible to prevent water mixing between the choice chambers (Stol *et al.*, 2013). Furthermore, it is beneficial if the height and the diameter of the passage can be adjusted to accommodate to the lifestyle of the animal, e.g. having the passage in the middle of the water column for species occupying the water column and near the bottom for benthic species, which will minimize water exchange between the two choice chambers and provide an easy passing between the choice chambers for the animal.

Systems where flow-through is used to control the environmental variable (Frank, 1971; Gregory and Anderson, 1984; Serrano *et al.*, 2010; Christensen and Grosell, 2018) need for an overflow. This overflow can both be placed in choice chambers and in the mixing chamber (Serrano *et al.*, 2010; Christensen and Grosell, 2018). However, having the overflow in the choice chambers will create better mixing of the water, which is added to the mixing chambers, and will thereby reduce water use.

# Video camera and backlight illumination

The video camera should be placed centrally above the shuttle-box in a sufficient distance to cover both choice chambers. Tracking in the areas where the vertical walls are visual are problematic due to minimal contrast difference with the animal, and these areas therefore typically constitute undetectable areas (Figure S2). Choosing the appropriate camera lens type and having a proper distance of the camera to the setup will minimize the problem with undetectable areas. A camera with a telephoto lens placed in good distance from the setup will minimize the amount of the vertical walls in the video recording and is therefore preferrable be preferred. On the contrary, a wide-angle lens cameras placed close to the setup will render more of the vertical walls visible (Figure S2), and hence should be avoided. It is also important to ensure that the setup takes up as much space within the field of view as possible but without any portions of the choice chamber outside the field of view. The maximizes the amount of pixels occupied by the choice chamber flooring, and the fish, and so optimizes tracking conditions. It should be noted, that having an elongated passage between the choice chambers emphasizes the issue of visible vertical walls, as the inner wall parts of the choice chambers will also become undetectable areas. It is therefore strongly recommended to use telephoto lens cameras to minimize undetectable areas in the setup, and having as short a passage between the choice chambers as possible. A mirror can be installed at a 45° angle to increase the distance to a horizontally placed camera (Habary *et al.*, 2016).


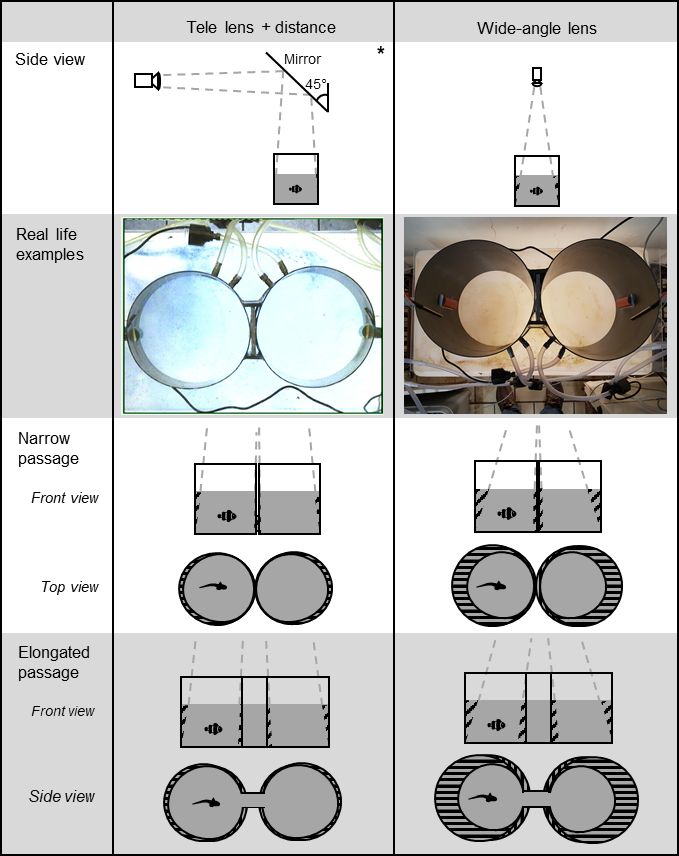


*Figure S2: Camera placement and the effect on lens type on tracking and undetectable areas. Grey dashed lines represents the camera viewing angles, and dashed areas indicate areas that are undetectable for the camera. * if the experimental room’s ceiling is high enough, a 45° angle mirror may not be necessary.*

Backlight illumination from underneath the setup provides good contrast and thereby increase tracking precision and accuracy. Infrared lighting is beneficial as it does not cause light to stress the animal (e.g. Serrano *et al.*, 2010; Herbert *et al.*, 2012; Stol *et al.*, 2013; Habary *et al.*, 2016). Using infrared lighting can also prevent faulty tracking during night (Nielsen and McGaw, 2016; Macnaughton *et al.*, 2018), and enable studying diurnal rhythms in environmental preference ranges (e.g. (Medvick and Miller, 1979; Staaks *et al.*, 1999; Serrano *et al.*, 2010).

# Experimental control and data acquisition

Experimental software for experimental control can, as for the physical setup, be bought commercially for around €5,000 for one license ([www.loligosystems.com](http://www.loligosystems.com), as of January 2020) or be custom made as in (Schurmann and Steffensen, 1994; Petersen and Steffensen, 2003; Tattersall *et al.*, 2012). The first step of computerized experimental control is transferring the video recording of the animal onto a computer with a frame-grabber if the signal is analog, or directly *via* USB-connection if the signal is digital (Schurmann and Christiansen, 1994; Petersen and Steffensen, 2003; Tattersall *et al.*, 2012; Stol *et al.*, 2013) (Figure S3). The environmental variable measurements are transferred to the computer either digitally through a USB-connection or *via* an analog-to-digital-converter (e.g. Measurement Computing USB-1208LS; see details on this device on [www.researchgate.net/project/Steffensen-shuttle-box-for-temperature-preference-how-to-build-and-run-it](http://www.researchgate.net/project/Steffensen-shuttle-box-for-temperature-preference-how-to-build-and-run-it)). The second step consists in real-time tracking of the animal. Only a few non-commercial software packages for real-time tracking of animals exist, such as ICfish (Tattersall *et al.*, 2012; Skandalis *et al.*, 2020) and SwisTrack (Lochmatter *et al.*, 2008). For shuttle-box setups, we have experience with using SwisTrack, which is freely available (<https://github.com/d28b/swistrack> ). Although SwisTrack has certain limitations, such as not yet being able to run on HD USB cameras, the built-in options are perfect for shuttle-box experiments (see instruction to SwisTrack on [www.researchgate.net/project/Steffensen-shuttle-box-for-temperature-preference-how-to-build-and-run-it](http://www.researchgate.net/project/Steffensen-shuttle-box-for-temperature-preference-how-to-build-and-run-it)). However, SwisTrack is currently being updated to enable use of HD USB cameras (Pers. Comm. T. Lochmatter. Custom tracking software can also be created using Python or R with OpenCV (e.g. Jolles, 2020), yet this requires some level of programming skill and time. The tracked data can be either written to a file or transmitted *via* a TCP port.

The third step is to track the position of the fish along with the environmental level of the two choice chambers, which is done in an experimental control and data acquisition software module (programmable in e.g. Labtech Notebook and Labtech Control, DaisyLab, LabView, or Python). Here, the following parameters should be given: the x-y coordinate ranges of the choice chambers to identify the position of the animal and hence when e.g. heating or cooling should be turned on or off, the choice chamber difference and the hysteresis for the system. Activation of counter-regulation should also be defined to prevent the environmental difference between the tanks becomes too low. It may be necessary to set the threshold for counter regulation slightly lower than the choice chamber difference to avoid excessive counter-regulation. If applying a maximum change rate this should also be defined here. Environmental levels may also have an upper and lower limit if deemed necessary (Christensen and Grosell, 2018). Information on the environmental level in each choice chamber and the spatial position of the animal (choice chamber occupancy and x-y coordinates for activity analyses) is then written at an appropriate frequency (e.g. 2 Hz) to a data storage file, e.g. *.txt. The experimental control and data acquisition module acts through a relay to control the experiment (Measurement Computing USB-1208LS + a custom made relay or a USB relay such as a Cleware USB Switch 4, (<http://www.cleware.de>).


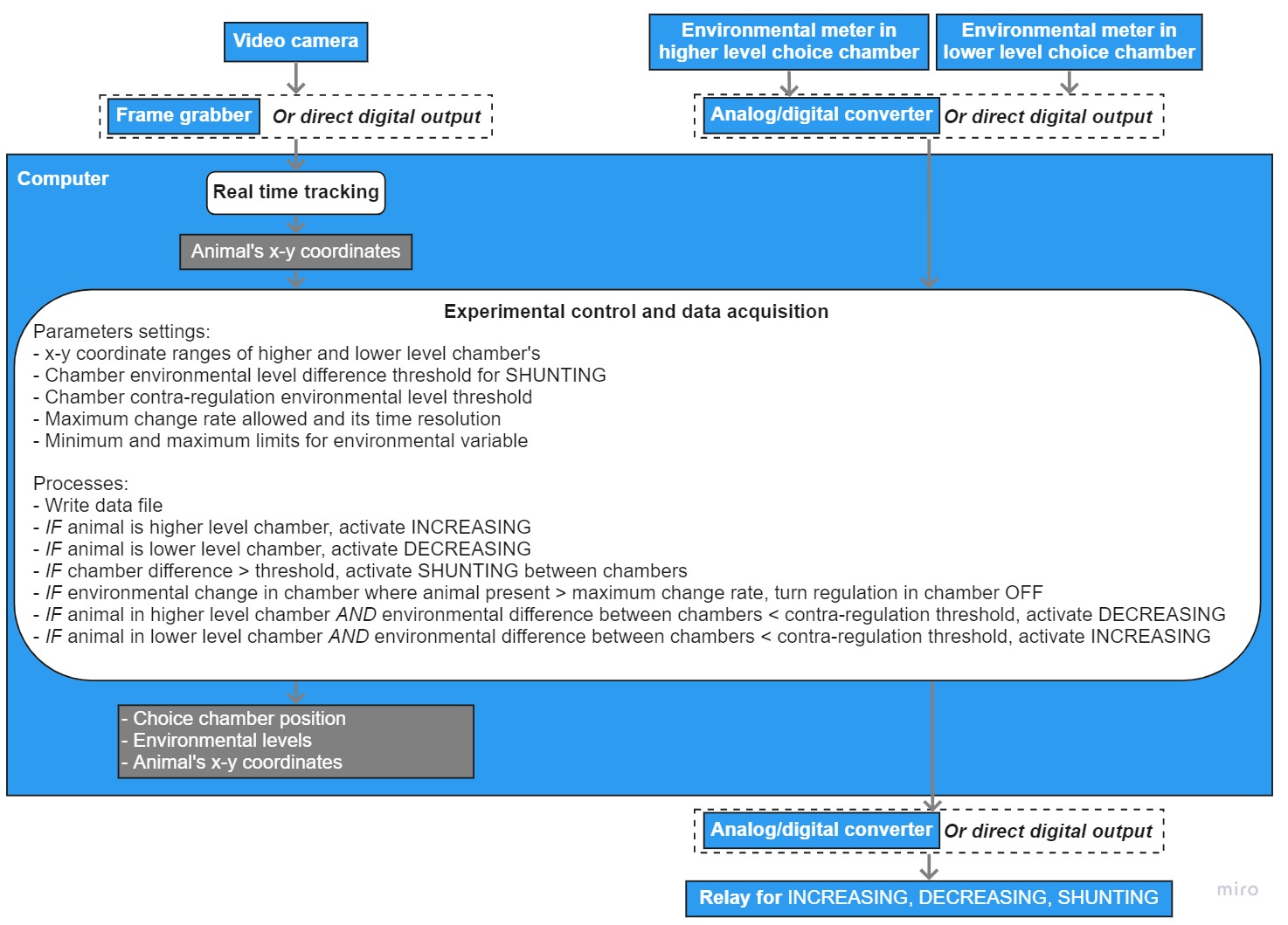


*Figure S3. General components of experimental control and data acquisition. Blue square boxes represents hardware components, grey arrows show output pathways, white rounded squares indicates software, and grey squares are file outputs.*

# Water dynamics

The shuttle-box should be placed as level as possible to easily enable equal water flow in the system, minimize gravitational mixing by the water in the choice chambers, and minimize potential chamber bias due to different water levels. Water circulation in the system is maintained by pumping water into the mixing chambers and relying on gravitation to move water back to the choice chambers. Having the bottom of the mixing chambers approximately at the same level as the bottom of choice chambers is a good rule of thumb to establish a pressure head without spilling over.

Water circulation can be adjusted by using properly sized pumps, or by using clamps to adjust water flow (Christensen and Grosell, 2018). In our experience, Eheim Universal pumps (EHEIM GmbH & Co. KG., Deizisau, Germany) are durable and come in a variety of sizes. The choice chamber’s water currents should be equal to most effectively maintain water separation, and sufficient to properly mix the water within the choice chambers, without causing turbulent flow or inducing exhaustive swimming of the animal. The efficiency of mixing within each chamber and water separation between the choice chambers can be assessed by adding colored dye to one mixing tower and follow the mixing properties of the system over time (Tietze and Gerald, 2016). In the case of temperature preference setups, a thermal camera can be used to test system water mixing and separation (Figure S4). As the water is more well mixed in the choice chamber than in the mixing chamber, placing the sensors between the mixing chamber outlet and the choice chamber inlet or in the choice chamber itself will provide the most stable measurements (Figure S1).


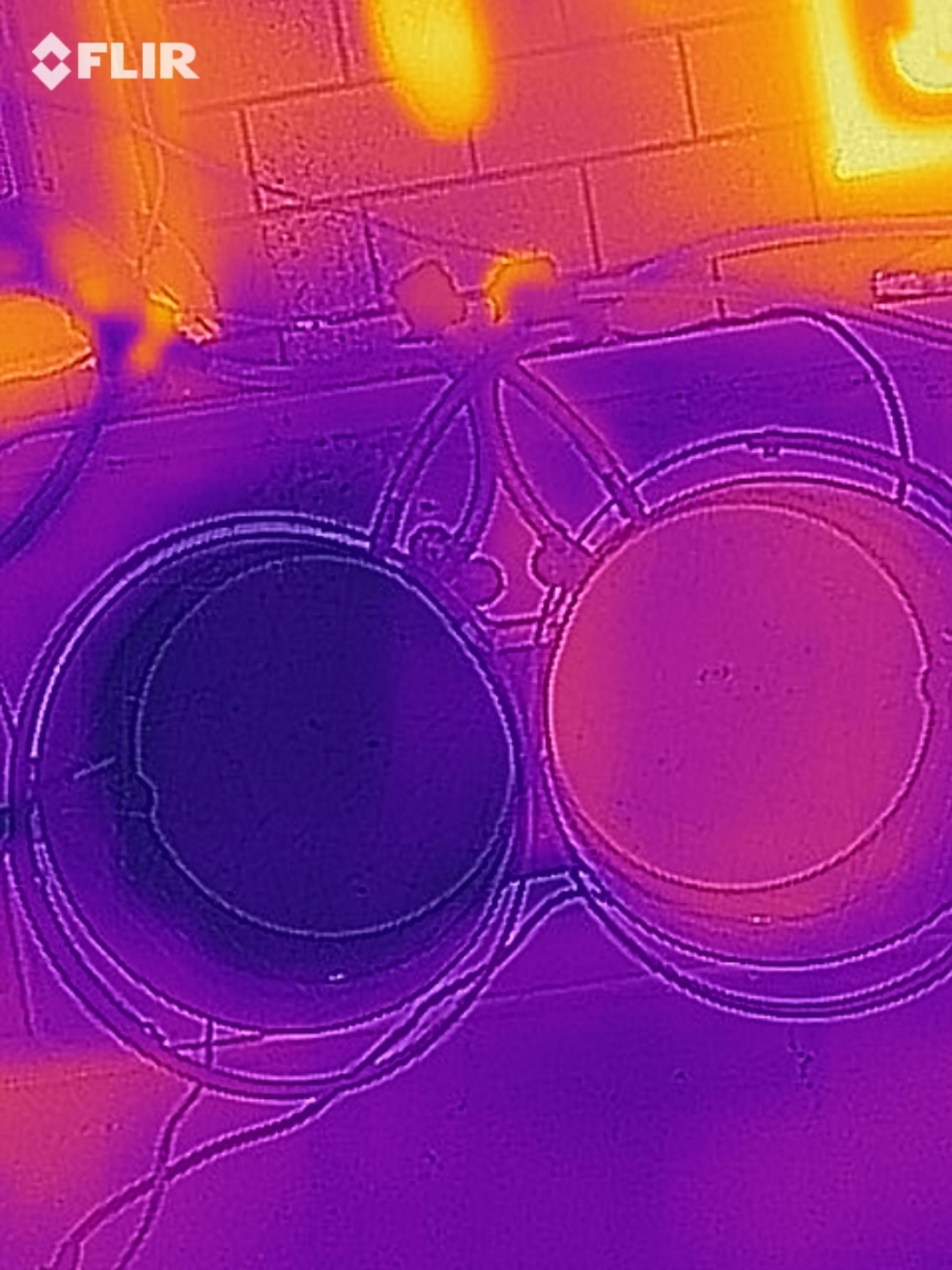


*Figure S4. Thermal camera picture to test the mixing and separation within a temperature shuttle-box. Photo by John F Steffensen.*

# Temperature

In temperature shuttle-boxes, water temperature should naturally be measured with high precision and accuracy. However, details on which temperature sensors are used are rarely given. We have used both PT100 sensors and thermo-couplers, which are precise, accurate, durable sensors with low drift. A good and relatively cheap solution is to use programmable LED indicator model 5714 with analog output from PR-Electronics (PR electronics, Rønde, Denmark) with a 4-wire PT-100 temperature sensor – for both choice chambers (find instructions on how to construct this device on [www.researchgate.net/project/Steffensen-shuttle-box-for-temperature-preference-how-to-build-and-run-it](http://www.researchgate.net/project/Steffensen-shuttle-box-for-temperature-preference-how-to-build-and-run-it)). This instrument is reliable and will show the temperature in the choice chambers even if the computer is turned off.

Temperature is usually regulated on the same body of water, which is achieved by activating pumps that lead water from the mixing tanks through heat exchangers placed in either a heating or a cooling bath. Controlling temperature by activating rod heaters placed in the mixing chamber will cause overshooting and is therefore not recommendable. Titanium heaters are usually efficient, sturdy, and relatively cheap to heat heating baths. In our experiences, ceramic and glass heaters can crack when handled and thus provide a safety hazard, while stainless steel heaters tend to corrode even though they are stainless steel. A simple, but laborious solution to chilling the cooling bath is to regularly add ice to the cooling bath (Harman *et al.*, 2020), which comes with a risk of disturbing the experimental animal in the process. Having chillers as cooling baths or chilling cooling baths with aquatic chillers are much more recommendable, but proper chillers with the correct cooling capacity can be costly. Furthermore, cooling is less effective than heating, and thus more power consuming per temperature change. Proper cooling capacity is especially important if the system temperatures should be able to approach the freezing point efficiently. When adding heaters and chillers, one should generally be sure not to get too close to the power output limit of wall plugs to prevent blowing fuses in the middle of experiments.

Bear in mind that the heat exchange with the ambient room is not taken into account in the supplementary “Heating-cooling power model”. This means, that if the experimental room is 10°C, cooling will be more efficient when the system water is above this temperature and lower when it is below, and vice versa for heating. Therefore, the needed heating/cooling power calculated above must be seen in relation to room temperature, and more power must be added if the system needs to be able to reach temperatures departing substantially from room temperature.

# Carbon dioxide

CO_2_ is added to the water is by gas infusing in the mixing chambers and removed by aeration. The partial pressure of CO_2_ (pCO_2_) can be measured directly with an infrared CO_2_ analyzer, or indirectly with a pH-electrode due to the bicarbonate dissociation equilibrium. As the bicarbonate dissociation equilibrium is dependent on water hardiness, calibration curves between ambient pCO_2_ level and water pH must first be established on the water used for experimenting (Kates *et al.*, 2012; Cupp *et al.*, 2017; Fredricks *et al.*, 2020). Pure CO_2_ gas can be injected to the water and automatically controlled via a feed-back from a pH-electrode. CO_2_ can also be added as a gas mixture from a gas mixing pump. Gas mixing pumps are expensive, but sturdy, and we have good experience with pumps from Wösthoff (Wösthoff Messtechnik GmbH, Bochum, Germany).

# Oxygen

A range of meters can be used to measure O_2_ level in water, but fiber optic meters are much more precise and stable over time than polarographic or galvanic sensors (Klimant *et al.*, 1995). Note that oxygen water solubility is temperature and pressure dependent and needs to be carefully compensated for (Garcia and Gordon, 1992). As stated in the main manuscript, it is not recommendable to inject pure oxygen to obtain normoxic levels.

O_2_ level is normally increased simply by aeration and decreased by infusing nitrogen gas to strip the water of O_2_ (Cook *et al.*, 2011; Herbert *et al.*, 2012; Nati *et al.*, 2018). In Borowiec *et al.* (2018), which was the only dynamic O_2_ shuttle-box study that was found in the literature review, O_2_ level was upregulated by infusing pure O_2_ gas. Unless the study is specifically designed to study hyperoxia, increasing O_2_ level by infusing pure O_2_ gas is not recommended, as this may cause simultaneous accumulation of CO_2_ in the water (Steffensen and Lomholt, 1988). Hypoxia and hypercapnia have interactive effects on aquatic animal physiology (Cruz-Neto and Steffensen, 1997), which may well affect their preference and avoidance of O_2_ level. Increasing O_2_ level by aeration is therefore always recommended, as it strips the water of dissolved CO_2_.

# Salinity

Salinity can be measured in high resolution with conductivity meters that can automatically temperature compensate. We have had good experiences with meters from the WTW ProfiLine series ([www.wtw.com](http://www.wtw.com)): the calibration of these meters are stable and usually lasts for an extended duration. As conductivity is measured through a small electric current salinity meters are sensitive to electrical interference, which may occur when having two conductivity meters connected to the same computer. The issue of electrical interference on the salinity meters can be eliminated with galvanic isolators between the meters and the computer, e.g. the USB2ISO (Electronics shop; www.electronics-shop.dk) (Christensen and Grosell, 2018).

High water usage is a major limitation to the system if a constant supply of suitable water is not at hand, or the costs for supplying the water is unfeasible. The asymptotic salinity limits could be expanded by using deionized water and salt enriched sea water as inflow water, but these are expensive solutions. In theory, shuttle-boxes can regulate salinity on the same water by using a reverse osmosis (RO) device, such as the ones used to generate fresh water from sea water on small boats and yachts (Figure S5). This system should be constructed with an overflow reservoir, from which RO device should filtrate the water, return the high saline effluent (of which there will be the highest volume) to the overflow reservoir, while delivering the low saline effluent to an adjacent tank; these two fractions are used as high saline and low saline reservoirs for the system, respectively. An overflow in the low saline tank will prevent the tank from overflowing and the high saline tank from emptying in the occasion that the animal occupies the high saline choice chamber for long time periods. The low saline effluent from RO has very low ion content, and will thus enable effective regulation of the system down towards fresh water salinity. This would both limit water use in salinity shuttle-box systems and enable the system to efficiently reach high and low salinity levels. However, such a system has never been build.

*
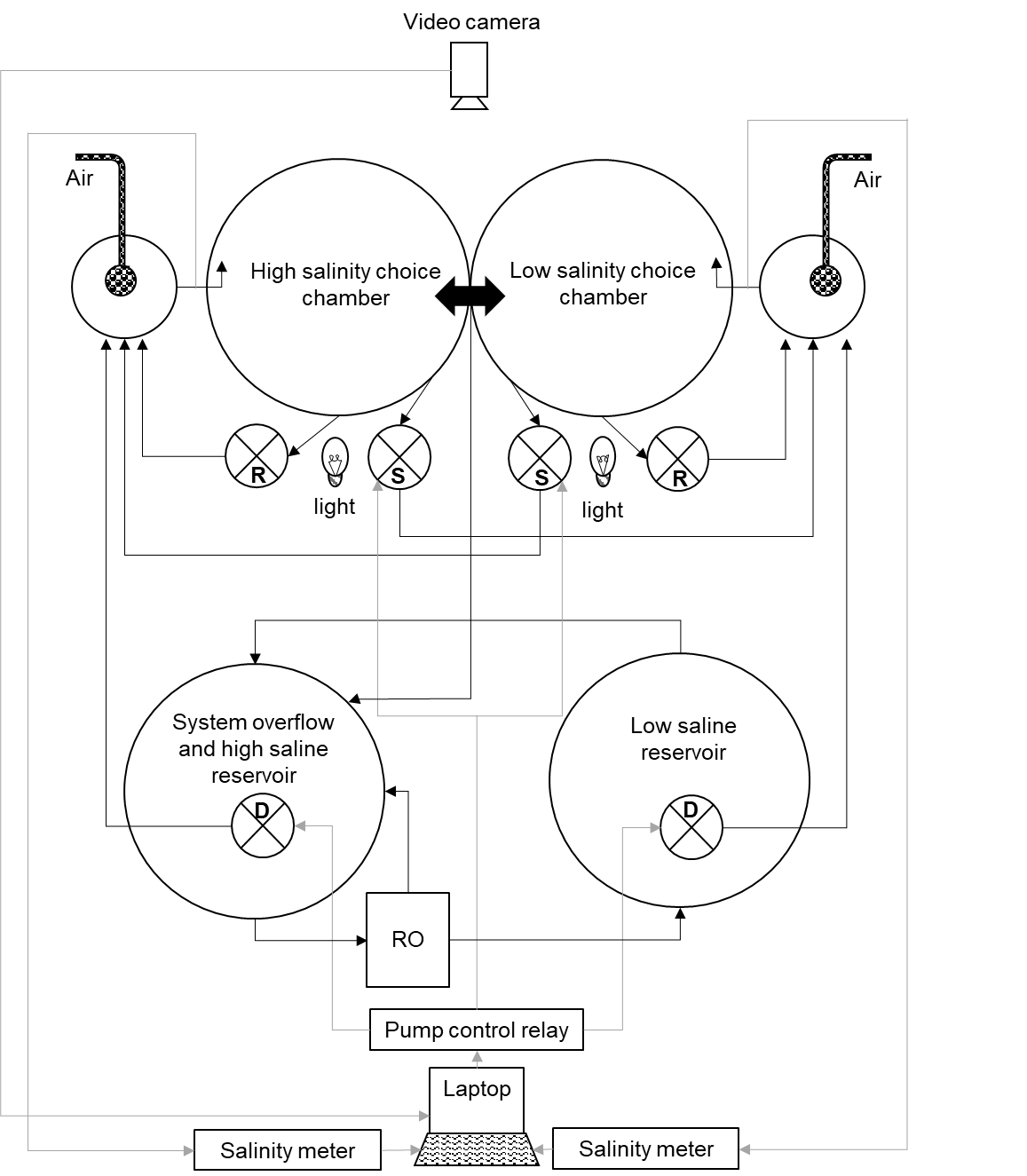
*

*Figure S5: Shuttle-box setup where salinity can be controlled on the same body of water with a reverse osmosis device (RO). White arrows show water movement and direction, dashed lines the water level. “R” denotes recirculation pumps, and passage between the choice chambers is shown by a black arrow. Dosage pumps (D) are used to control the salinity in the system. Shunt pumps (S) are used to maintain a fixed environmental difference between the choice chambers. Grey arrows symbolizes signaling pathways for experimental control and data acquisition.*

# Multiple variables

The studies in which manipulation of multiple variables occurs, certain modifications of the standard shuttle-box set up may be necessary. For instance, for feeding during trials, installing a feeding device may be necessary (e.g. as in Larsson, 2005). In studies using shelters or structural environment as additional test variable, these structures may hide the study animal and thus obstruct tracking. Nay *et al.* (2020) solved this by adding a mask to the structure areas in their tracking software and programming it to use the last detected position when the animal entered the structure. Nielsen and McGaw (2016) solved the issue of tracking when their animal was in a shelter by drilling holes in it to make it semi-transparent. For experiments on environmental preference at different light levels, different light sources can simply be used, as in Fredriks *et al.* (2020), or by constructing a two axis, four chamber shuttle-box as in Reynolds and Casterlin (1976) and Reynolds (1977). For studying trade-offs of environmental preference with sociability, conspecifics can be places in a transparent compartment within the choice chambers, which can then be masked in the tracking (Cooper *et al*., 2018; Borowiec *et al*., 2018; Tucker and Suski, 2019). A similar solution as the one for sociability could in principle be used for studying trade-off between environmental preference and predator presence. Chemical agents, such as predator cues, can be added in systems that run on flow-through regulation, e.g. Gregory and Anderson (1984) and Tietze and Gerald (2016). In studies on effects of gaseous agents on environmental preference, such as hypoxia (induced with nitrogen) or hydrogen sulfur, these can be induced by gassing in the mixing chambers (Schurmann *et al*., 1991, Schurmann and Steffensen, 1992, Enders *et al.*, 2019: Skandalis *et al*., 2020).

# References

Bevelhimer MS (1996) Relative importance of temperature, food, and physical structure to habitat choice by smallmouth bass in laboratory experiments. *Trans AM Fisher Soc* 125: 274–283.

Borowiec BG, O’Connor CM, Goodick K, Scott GR, Balshine S (2018) The preference for social affiliation renders fish willing to accept lower O_2_ levels. *Physiol Biochem Zool* 91: 716–724.

Cook DG, Wells RMG, Herbert NA (2011) Anaemia adjusts the aerobic physiology of snapper (*Pagrus auratus*) and modulates hypoxia avoidance behaviour during oxygen choice presentations. *J Exp Biol* 214: 2927–2934.

Christensen EAF, Grosell M (2018) Behavioural salinity preference of juvenile yellow perch *Perca flavescens*: salinity preference of *Perca flavescens*. *J Fish Biol* 92: 1620–1626.

Cooper B, Adriaenssens B, Killen SS (2018) Individual variation in the compromise between social group membership and exposure to preferred temperatures. *Proc R Soc B* 285: 20180884.

Cruz-Neto AP, Steffensen JF (1997) The effects of acute hypoxia and hypercapnia on oxygen consumption of the freshwater European eel. *J Fish Biol* 50: 759–769.

Cupp A, Tix J, Smerud J, Erickson R, Fredricks K, Amberg J, Suski C, Wakeman R (2017) Using dissolved carbon dioxide to alter the behavior of invasive round goby. *MBI* 8: 567–574.

Enders EC, Wall AJ, Svendsen JC (2019) Hypoxia but not shy-bold phenotype mediates thermal preferences in a threatened freshwater fish, *Notropis percobromus*. *J Therm Biol* 84: 479–487.

Frank LH (1971) A technique for measuring thermoregulatory behavior in the fish. *Behav Res Meth Instru* 3: 250–250.

Fredricks K, Tix J, Smerud J, Cupp A (2020) Laboratory trials to evaluate carbon dioxide as a potential behavioral control method for invasive red swamp (Procambarus clarkii) and rusty crayfish (Faxonius rusticus). *MBI* 11: 259–278.

Garcia HE, Gordon LI (1992) Oxygen solubility in seawater: Better fitting equations. *Limnol Oceanogr* 37: 1307–1312.

Gregory MA, Anderson PD (1984) A modified electronic shuttlebox for joint thermoregulatory and toxicological studies. *Can J Zool* 62: 1950–1953.

Habary A, Johansen JL, Nay TJ, Steffensen JF, Rummer JL (2016) Adapt, move or die - how will tropical coral reef fishes cope with ocean warming? *Glob Change Biol* 23: 566–577.

Harman AA, Fuzzen M, Stoa L, Boreham D, Manzon R, Somers CM, Wilson JY (2020) Evaluating Tank Acclimation and Trial Length for Shuttle-box Temperature Preference Assays (preprint). Physiology.

Herbert NA, Goodman M, Kunzmann A (2012) The low O _2_ avoidance strategy of the Cape silverside *Atherina breviceps* (Teleostei). *Marine and Freshwater Behaviour and Physiology* 45: 199–208.

Jolles J (2020) pirecorder: Controlled and automated image and video recording with the raspberry pi. *JOSS* 5: 2584.

Kates D, Dennis C, Noatch MR, Suski CD (2012) Responses of native and invasive fishes to carbon dioxide: potential for a nonphysical barrier to fish dispersal. *Can J Fish Aquat Sci* 69: 1748–1759.

Klimant I, Meyer V, Kühl M (1995) Fiber-optic oxygen microsensors, a new tool in aquatic biology. *Limnol Oceanogr* 40: 1159–1165.

Larsson S (2005) Thermal preference of Arctic charr, *Salvelinus alpinus*, and brown trout, *Salmo trutta* – implications for their niche segregation. *Environ Biol Fish* 73: 89–96.

Lochmatter T, Roduit P, Cianci C, Correll N, Jacot J, Martinoli A (2008) SwisTrack - A Flexible Open Source Tracking Software for Multi-Agent Systems. In: 2008 IEEE/RSJ International Conference on Intelligent Robots and Systems. Presented at the 2008 IEEE/RSJ International Conference on Intelligent Robots and Systems, IEEE, Nice, pp 4004–4010.

Nay TJ, Johansen JL, Rummer JL, Steffensen JF, Pratchett MS, Hoey AS (2020) Habitat complexity influences selection of thermal environment in a common coral reef fish. *Conserv Physiol* 8: coaa070.

Macnaughton CJ, Kovachik C, Charles C, Enders EC (2018) Using the shuttlebox experimental design to determine temperature preference for juvenile Westslope Cutthroat Trout (Oncorhynchus clarkii lewisi). *Conservation Physiology* 6. doi:10.1093/conphys/coy018

Mccauley RW, Elliott JR, Read LAA (1977) Influence of acclimation temperature on preferred temperature in the rainbow trout *Salmo gairdneri*. *Trans Am Fisher Soc* 106: 362–365.

Medvick PA, Miller JM (1979) Behavioral thermoregulation in three Hawaiian reef fishes. *Environ Biol Fish* 4: 23–28.

Myrick CA, Folgner DK, Cech JJ (2004) An annular chamber for aquatic animal preference studies. *Trans Am Fisher Soc* 133: 427–433.

Nati JJH, Lindström J, Yeomans W, Killen SS (2018) Physiological and behavioural responses to hypoxia in an invasive freshwater fish species and a native competitor. *Ecol Freshw Fish* 27: 813–821.

Nielsen TV, McGaw IJ (2016) Behavioral Thermoregulation and Trade-Offs in Juvenile Lobster *Homarus americanus*. *The Biological Bulletin* 230: 35–50.

Petersen MF, Steffensen JF (2003) Preferred temperature of juvenile Atlantic cod Gadus morhua with different haemoglobin genotypes at normoxia and moderate hypoxia. *Journal of Experimental Biology* 206: 359–364.

Reynolds WW (1977) Fish Orientation Behavior: An electronic device for studying simultaneous responses to two variables. *J Fish Res Bd Can* 34: 300–304.

Reynolds WW, Casterlin ME (1976) Locomotor activity rhythms in the bluegill sunfish, *Lepomis macrochirus*. *Am Midland Naturalist* 96: 221.

Schurmann H, Christiansen JS (1994) Behavioral thermoregulation and swimming activity of two arctic teleosts (subfamily gadinae)—the polar cod (Boreogadus saida) and the navaga (Eleginus navaga). *Journal of Thermal Biology* 19: 207–212.

Schurmann H, Steffensen JF (1992) Lethal oxygen levels at different temperatures and the preferred temperature during hypoxia of the Atlantic cod, *Gadus morhua* L. *J Fish Biol* 41: 927–934.

Schurmann H, Steffensen JF, Lomholt JP (1991) The influence of hypoxia on the preferred temperature of rainbow trout *Oncorhynchus mykiss*. *J Exp Biol* 157: 75–86.

Schurmann H, Steffensen JF (1994) Spontaneous swimming activity of Atlantic cod *Gadus morhua* exposed to graded hypoxia at three temperatures. *J Exp Biol* 197: 129–142.

Serrano X, Grosell M, Serafy JE (2010) Salinity selection and preference of the grey snapper Lutjanus griseus: field and laboratory observations. *Journal of Fish Biology* 76: 1592–1608.

Skandalis DA, Dobell CD, Shaw JC, Tattersall GJ (2020) Hydrogen Sulfide Exposure Reduces Thermal Set Point in Zebrafish (preprint). Physiology.

Staaks G, Kirschbaum F, Williot P (1999) Experimental studies on thermal behaviour and diurnal activity rhythms of juvenile European sturgeon (Acipenser sturio). *J Appl Ichthyol* 15: 243–247.

Steffensen JF, Lomholt JP (1988) Accumulation of carbon dioxide in fish farms with recirculating water. In: Fish Physiology, Fish Toxicology, and Fisheries Management. Environmental Research Laboratory Office of Research and Development US Environmental Protection Agency, Guangzhou, pp 157–161.Stol JA, Svendsen JS, Enders EC (2013) Determining the thermal preferences of Carmine Shiner (Notropis percobromus) and Lake Sturgeon (Acipenser fulvescens) using an automated shuttlebox. doi:10.13140/RG.2.1.1672.0245

Tattersall GJ, Luebbert JP, LePine OK, Ormerod KG, Mercier AJ (2012) Thermal games in crayfish depend on establishment of social hierarchies. *Journal of Experimental Biology* 215: 1892–1904.

Tietze SM, Gerald GW (2016) Trade-offs between salinity preference and antipredator behaviour in the euryhaline sailfin molly *Poecilia latipinna*: salinity preference *v.* predator avoidance. *J Fish Biol* 88: 1918–1931.

Tucker EK, Suski CD (2019) Presence of conspecifics reduces between-individual variation and increases avoidance of multiple stressors in bluegill. *Animal Behaviour* 158: 15–24.
