# Supplementary material for: Shuttle-box systems for studying preferred environmental ranges by aquatic animals
Source: Conserv Physiol. 2021 May 17;9(1):coab028. doi: 10.1093/conphys/coab028 (PMC8129825; doi:10.1093/conphys/coab028)
Supplement: suppl_data_coab028 [file suppl_data_coab028.zip › Shuttle-box blue-print.pdf]

NW isometric view

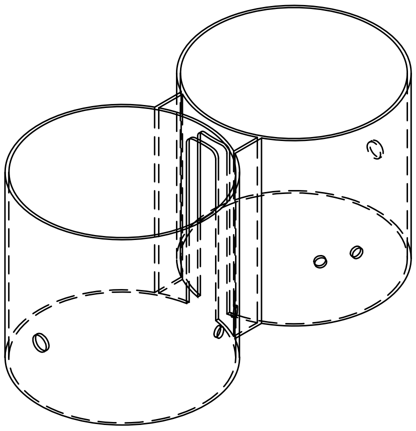

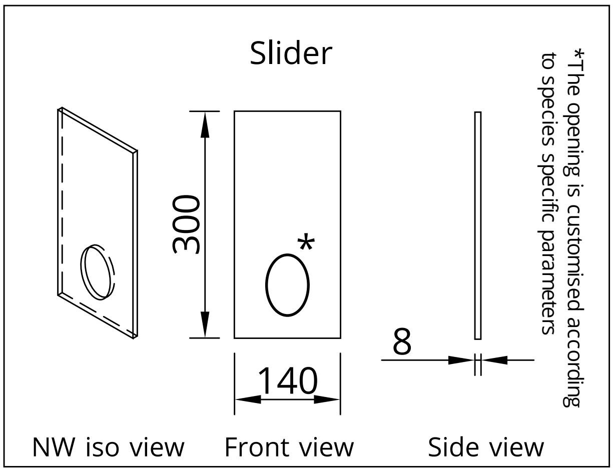

Top view

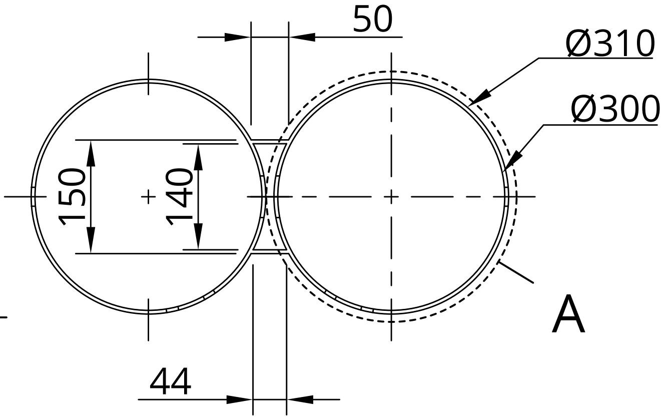

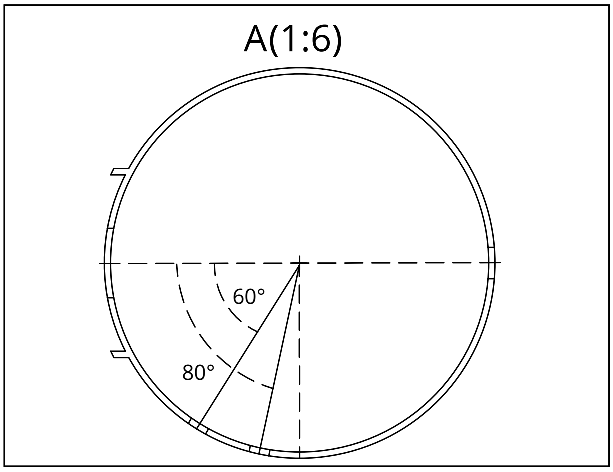

Side view

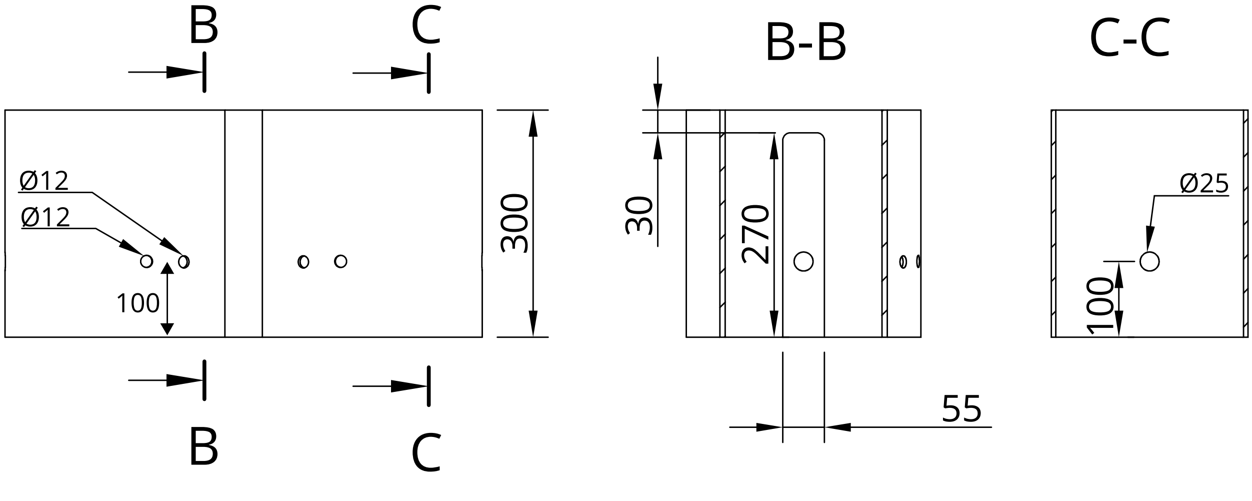

¤ Please note that all characteristics are mirrored along the plane separating the two preference chambers.

¤ All connectors for tubing are retrofitted.

Scale 1:10; Dimensions in mm

Diffuser

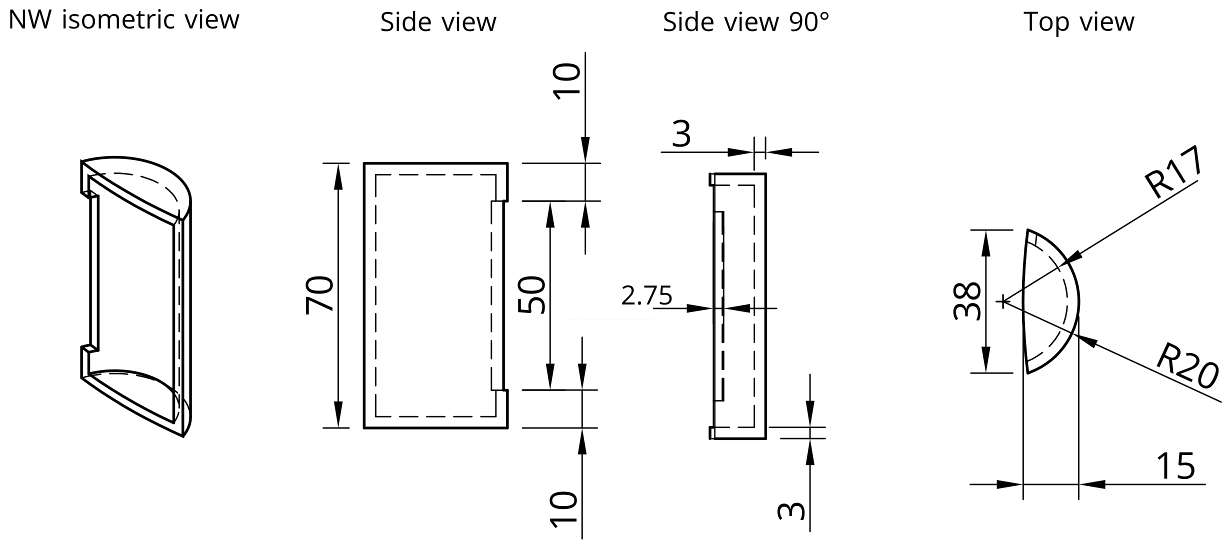

Scale 1:2; Dimensions in mm

Mixing Chamber

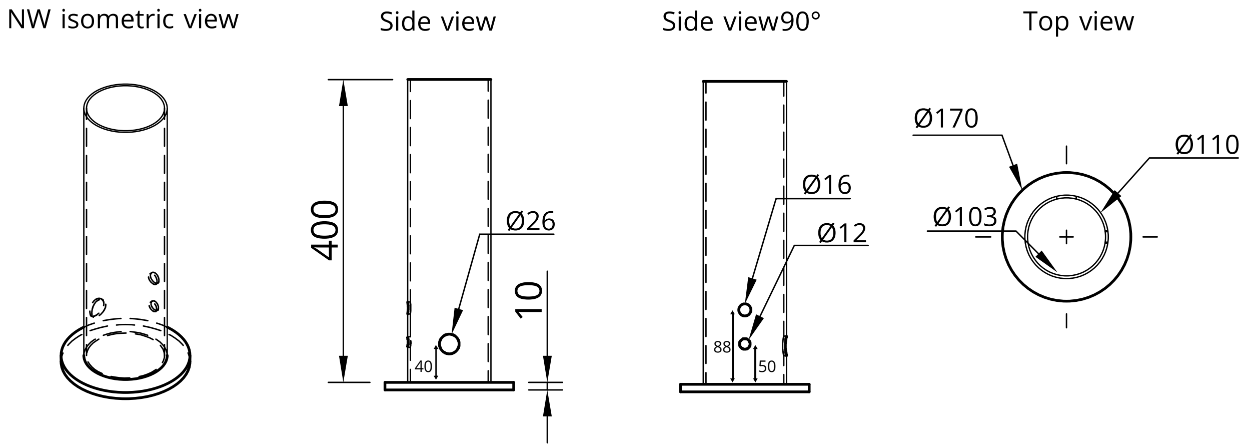

¤ All connectors fort ubing are retrofitted.

Scale 1:10; Dimensions in mm
